# Magnetic resonance imaging for adult idiopathic inflammatory myopathies: A scoping review of protocols, grading systems and applications

**DOI:** 10.1016/j.semarthrit.2025.152865

**Published:** 2025-11-07

**Authors:** Jessica A. Day, Daniel Brito de Araújo, Mickael Essouma, Edoardo Conticini, Lisa G. Rider, Daren Gibson, Adriana Maluf Elias, Claudia Saad Magalhães, Simone Appenzeller, Adam Schiffenbauer, Anneke J van der Koi, Siamak Moghadam-Kia, Vitor Tavares Paula, Julio Brandão Guimarães, Edoardo Marrani, Andrea Schwarz Doria, Samuel Katsuyuki Shinjo

**Affiliations:** a Environmental Autoimmunity Group, Clinical Research Branch, National Institute of Environmental Health Sciences, National Institutes of Health, Bethesda, MD, United States; b Department of Diagnostic & Interventional Radiology, Research Institute, The Hospital for Sick Children, University of Toronto, Toronto, ON, Canada; c Pediatric Rheumatology Unit, Children’s Institute, Hospital das Clínicas HCFMUSP, Faculdade de Medicina, Universidade de São Paulo, São Paulo, SP, Brazil; d Department of Neurology, Amsterdam UMC, University of Amsterdam, Amsterdam Neuroscience, Amsterdam, the Netherlands; e Pediatric Rheumatology Unit, Department of Pathology, Botucatu Medical School, São Paulo State University, Botucatu, São Paulo, Brazil; f Internal Medicine Department, Universidade Federal de Pelotas, Pelotas, Rio Grande do Sul, RS, Brazil; g Medical Imaging, Fiona Stanley Hospital, Murdoch, Australia; h Rheumatology Unit, Department of Medicine, Surgery and Neurosciences, University of Siena, Siena, Italy; i Rheumatology Unit, ERN-ReCONNET Center, Meyer Children’s Hospital IRCCS, Florence, Italy; j The Walter and Eliza Hall Institute of Medical Research, Melbourne, Victoria, Australia; k Royal Melbourne Hospital, Melbourne, Victoria, Australia; l The University of Melbourne, Melbourne, Victoria, Australia; m Universidade Federal de São Paulo, EPM-UNIFESP, São Paulo, Brazil; n Grupo Fleury Medicina e Saúde, São Paulo, Brazil; o Network of Immunity in Infection, Malignancy and Autoimmunity, Universal Scientific Education and Research Network, Yaounde, Cameroon; p Division of Rheumatology, Faculdade de Medicina FMUSP, Universidade de São Paulo, São Paulo, Brazil; q Division of Rheumatology and Clinical Immunology, Department of Medicine, University of Pittsburgh School of Medicine, Pittsburgh, PA, USA; r Department of Orthopedics, Rheumatology and Traumatology, University of Campinas, Campinas, SP, Brazil; s Musculokeletal radiologist. Radiology Department, Children’s Institute, Hospital das Clínicas HCFMUSP, Faculdade de Medicina, Universidade de São Paulo, São Paulo, SP, Brazil; t Hospital Israelita Albert Einstein, SP, Brazil

**Keywords:** Idiopathic inflammatory myopathies, Myositis, MRI, Magnetic resonance imaging

## Abstract

Magnetic resonance imaging (MRI) has emerged as a key non-invasive tool for the evaluation of idiopathic inflammatory myopathies (IIM); however, heterogeneity in techniques, protocols, and grading systemics impedes standardization. This scoping review systematically examined the MRI techniques, protocols, and grading systems reported in the adult IIM literature. A systematic search of PubMed, EMBASE, and Cochrane databases was conducted from 2000 to 2024 using keywords related to IIM and MRI. Studies involving adults with IIM who underwent MRI were screened and reviewed for inclusion. Forty-nine studies were included in the analysis, 13 of which evaluated whole-body MRI and 36 evaluated dedicated body-part MRI, collectively reporting data from 2810 IIM patients. A wide range of imaging protocols was observed with variations in scanner type, field strength, sequence combinations, and anatomical coverage. Semi-quantitative visual grading was the most commonly used assessment method (31/49, 63.2 %), with binary scoring in 23/31 and software-assisted or automated techniques in 8/31. Six studies used descriptive analysis alone. Inter-rater agreement was reported in 15 studies, with variable reliability observed for both muscle edema (intraclass correlation coefficient [ICC] range: 0.78–1.00; kappa range: 0.30–1.00) and replacement of skeletal muscle by fat (ICC range: 0.77–0.97; kappa range: 0.54–0.93). Several studies have reported that WB-MRI patterns correlate with clinical measures of disease activity and can discriminate between myopathic diseases and IIM subtypes. In summary, despite the clinical utility of MRI for IIM, significant methodological variability remains. Future research should focus on standardizing protocols and grading systems to enhance the consistency and reliability of MRI assessments for IIM.

## Introduction

Idiopathic inflammatory myopathies (IIM) are a heterogeneous group of multisystem autoimmune diseases that share a common predilection for skeletal muscle inflammation and extramuscular manifestations. These diseases are subdivided into dermatomyositis (DM), clinically amyopathic DM (CADM), polymyositis (PM), immune-mediated necrotizing myopathy (IMNM), anti-synthetase syndrome (ASyS), and inclusion body myositis (IBM) [1]. The diagnosis and classification of IIM have traditionally relied on a combination of characteristic clinical features, presence of myositis autoantibodies, and histological examination of the muscle or skin. Although most classification criteria for IIM omit imaging evaluation with magnetic resonance imaging (MRI) as a domain, this modality has been increasingly utilized as a noninvasive adjunct to aid diagnosis, guide muscle biopsy sites, assess disease severity, and monitor therapy and disease progression.

Historically, targeted muscle or dedicated body-part MRI (DBP-MRI) has been used in adults with muscle diseases. However, with newer sequences, coil developments, and the wider availability of MRI scanners, there has been growing interest in whole-body (WB) image characterization and phenotyping of patients with suspected or known IIM [2,3]. The major advantage of WB-MRI over DBP-MRI is that it provides a comprehensive single assessment of the total burden and distribution of inflammation across the appendicular and axial muscles, fascia, and subcutaneous tissues, potentially allowing the development of a “global” disease measure. Outside of the primary diagnostic aim, viscera and bone are included in the images, and incidental findings are occasionally discovered [4]. The distribution of muscle and fascial involvement may aid in discriminating against myositis subtypes with distinct patterns. Muscle site selection for diagnostic invasive muscle biopsy sampling and inflammation within deeper-seated muscles is difficult to examine clinically (e.g., the trunk and paraspinal muscles), and a comprehensive estimate of the total muscular inflammatory burden may be facilitated with this technique.

A significant limitation of cross-site research and MRI integration into diagnostic pathways is the absence of universally accepted and validated protocols for IIM assessments [2,5]. Although qualitative and semi-quantitative scoring systems quantifying muscle inflammation and replacement of skeletal muscle by fat (‘fatty replacement’) have been developed for myopathies, they lack standardization and validation for IIM subtyping. Moreover, these grading systems typically rely on subjective visual assessments and are constrained by the availability of disease-specific subspecialists. The heterogeneity of MRI scanning practices across geographical domains presents an additional challenge. It is difficult to compare results between centers because patient positioning, coil use, and scan parameters are not standardized.

Therefore, the aims of this scoping review are to (*i*) provide an overview of the various adult WB-MRI and DBP-MRI techniques, protocols, and grading systems reported in the IIM literature; (*ii*) identify potential methodological limitations; (*iii*) analyze whether the available MRI scoring systems have undergone methodological evaluation concerning their clinimetric properties; and (*iv*) propose future directions and research to address knowledge gaps.

## Materials and methods

A working group of the International Myositis Assessment and Clinical Studies Group (IMACS) was assembled to review the literature on MRI in adults with IIM. PubMed (Medline), EMBASE, and Cochrane databases were searched electronically with the assistance of a medical librarian from January 1, 2000, to June 1, 2024. We used keywords and Medical Subject Headings (MeSH) terms related to IIM and MRI, as defined in protocol [3]. The reference lists of the original and review articles were manually searched for potentially relevant studies. Ten investigators screened the full text for potential inclusion. Each publication was independently screened by two investigators and evaluated by a third investigator if consensus was not reached [3].

### Inclusion and exclusion criteria

We included studies that investigated the use of WB-MRI and DBP-MRI in the evaluation of adult IIM with qualitative, semi-quantitative, or quantitative assessments of subject muscle signal changes. Non-inflammatory forms of myopathy were excluded unless part of the cohort included inflammatory myopathies. Studies that did not report a characterization or quantification system for radiological muscular changes were excluded. Studies that included both adults and children were included if the mean/median age of the population was ≥18 years, as previously used as a cutoff inclusion criterion for pediatric/adult studies [6]. WB-MRI was defined as a single MRI scan capturing the entire span from the head or neck to the toes, whereas DBP-MRI was defined as one or two distinct and selected body regions within a single session, including the shoulder and/or pelvic girdle/thighs. Abstracts, case reports with fewer than five patients, reviews, comments, and letters to the editors were excluded, as defined in the protocol [3].

### Data extraction, synthesis, and analysis

We extracted data regarding the study type, patient characteristics, MRI protocols, and grading systems using a pre-designed data extraction sheet. Each study underwent data extraction by two independent investigators and was cross-checked by the convener of the Task Force, a radiologist-epidemiologist (ASD). A qualitative synthesis of the extracted data was performed. Means, standard deviations (SD), medians, and (minimum – maximum, or interquartile [IQR]) ranges were used for descriptive analysis.

### Ethical approval

Not required. This study was a systematic review of previously published studies and did not involve human participants or new data collection.

## Results

**Fig. 1.**
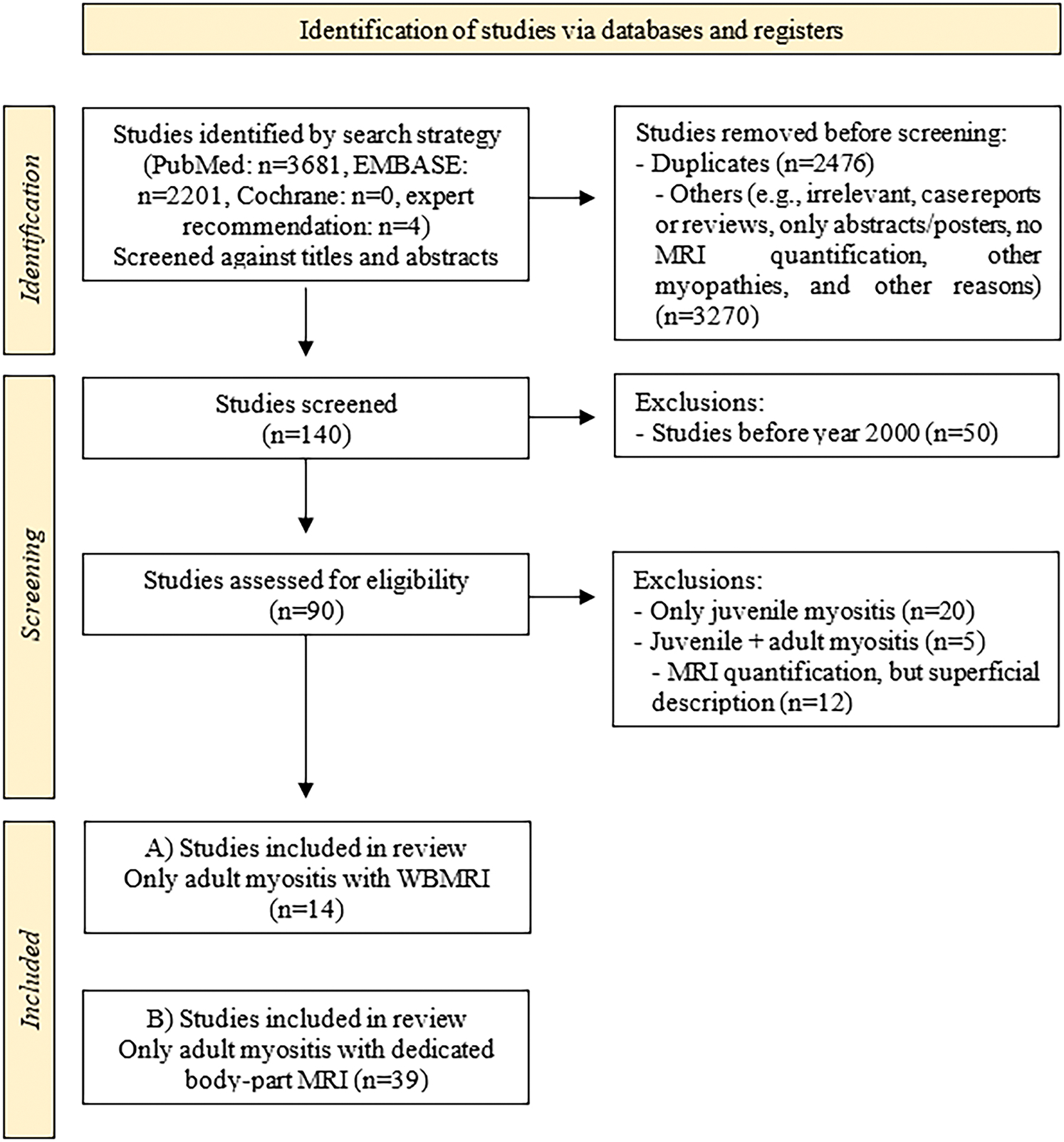


Our search initially identified 5886 studies, with 2476 duplicates removed. In the screening phase, we removed 3270 articles that were excluded based on the title and abstracts, primarily due to the topic and design criteria (Fig. 1). Of the remaining 140 articles, 50 studies published before 2000 were excluded, as recent technical manufacturer-driven MRI advances would not have allowed an accurate cross comparison of imaging data. We reviewed 86 full-text articles, excluding 37 based on the prior inclusion/exclusion criteria mentioned in this scoping review. This resulted in 14 WB-MRI studies [4,7–19] and 39 DBP-MRI studies [20–58] being included in the review.

### A) WB-MRI studies

#### Study characteristics

Eleven WB-MRI studies evaluated cohorts comprising a mixture of IIM subtypes [4,7–9,12–19], whereas one study evaluated only IBM [10] and two evaluated only DM/CADM cohorts [11,13]. The most common subtypes of adult IIM described in the WB-MRI literature, ranked by the pooled sum of subjects, were DM (*n* = 203), IBM (*n* = 104), PM (*n* = 78), IMNM (*n* = 66), overlap myositis (OP) (*n* = 25), CADM (*n* = 17), inflammatory myopathy or PM-mitochondrial pathology (*n* = 17), PM/ASyS/OP (*n* = 11), ASyS (*n* = 8), anti-mitochondrial autoantibody (AMA) myositis (*n* = 2), and cancer-associated myositis (*n* = 1). All included studies were observational, with prospective [7,8,16], retrospective [4,12–14], or cross-sectional [9,15,17–19] designs. The primary study samples ranged from eight to 129 subjects with IIM (Table 1). All studies evaluated the role of WB-MRI in the diagnosis or characterization of muscle abnormalities in IIM, with 13 reporting muscle edema or inflammation [4–7,10–18], nine reporting fatty replacement [4,8,11,12,14,16–19], and four reporting muscle atrophy or quantifying muscle volumes or degree of atrophy [4,10,12,14]. Six studies also reported fascial edema or other extramuscular features [4,7,9,11,13,15]. Only two studies [11,13] examined newly diagnosed IIM alone, whereas 12 studies examined cohorts of patients with longstanding, treatment-initiated, or mixed disease durations [4,7–10,12,14–19]. In most studies, at least a proportion of the cohort was under therapeutic immunosuppression at the time of MRI. Two studies reported that the entire study population was not on immunosuppressive therapy at the time of imaging [7,13].

#### WB-MRI protocols

Various WB-MRI protocols and procedural approaches have been reported (Table 2). Eight studies utilized a 1.5T scanner [7,9,10–14,17], whilst six operated at 3T field strength [4,8,15,16,18,19]. One study reported the use of gadolinium [8]. The regions imaged were variably described (Table 2), with a single study comparing WB-MRI with a limited protocol that omitted the trunk [8]. Edge-of-field imaging generates artifacts, and imaging of the forearms was excluded in two studies because of concerns regarding poor image resolution, fat suppression, or peripheral field-of-view deterioration [8,15]. Two studies used short tau inversion recovery (STIR) sequences in isolation [7,13], eight used a combination of STIR and T1 sequences [4–6,10,11,14,15], one used only T2 sequences [16], and one only used T1 sequences [19]. One study evaluated the additional benefit of diffusion-weighted (DW) sequences [12], one used STIR/Fast Spin Echo (FSE) [17], and a single study used T1/FSE [18]. Ten studies included both axial and coronal slices [4–7,11,13,16,19], whereas four examined axial slices alone [10,12,17,18]. Repetition and echo times varied according to the MRI protocols of different studies (Table 2). The total acquisition time for the fastest studies involving STIR-only sequences ranged from 10 to 20 min. As expected, the total acquisition time tended to be longer when multiple additional sequences were used (15–60 min, depending on the sequence) (Table 2). The omission of trunk imaging in a solitary study reduced the average acquisition time to 18 min 24 s without affecting the diagnostic yields [8].

#### WB-MRI evaluation systems

##### Evaluation of muscle edema

Various scoring systems have been developed to assess muscle edema (Table 3). Six studies used semi-quantitative grading scales [8–11,15,16], and five used binary scoring (present/absent) of individual muscles or regions [4,13,14,17,18]. Three studies [8,12,16] used a grading scale previously developed for the assessment of WB-MRI in juvenile DM [59], whereas three studies [9,10,15] used grading systems initially devised for muscular dystrophy [60] and other neuromuscular disorders [61, 62]. The definitions used in the semi-quantitative grading systems varied (Table 3). In five studies, the total burden of muscle inflammation was quantified by summing the edema scores of individual muscle groups [8,11,13,15,16]. In one study, the mean muscle edema score was calculated by dividing the total score by the number of muscles evaluated [12].

##### Evaluation of fatty muscle replacement

Six studies evaluated fatty replacement using semi-quantitative grading [8,10,12,17–19] and referenced previously published grading scales. Two studies [16,17] used semi-quantitative grading scales developed for inherited neuromuscular disorders - Mercuri score [63] and Fischer score [64]. The Mercuri scoring system employs a 0- to 3-point scale that combines detailed descriptions and the percentage of muscle affected to determine the grade of fatty replacement. Two studies [8,10] referenced a system initially developed to evaluate fatty degeneration in rotator cuff musculature [65], whereas in the fourth study [12], the referenced grading scale was not elaborated upon in the cited study [66]. Three studies used binary scoring to evaluate fatty replacement (present/absent) [4,11,16].

##### Evaluation of fascial edema

One study evaluated fascial edema using a semi-quantitative 0–3 scale [11], whereas five studies evaluated fascial edema using binary scoring of muscles or regions (absent/present) [8,9,13,15,16]. Three studies summed up the fascial edema scores to evaluate the total burden of fascial involvement [11,13,15].

##### Evaluation of muscle atrophy

Muscle atrophy (volume loss) was assessed using a binary scale (present/absent) in one study that evaluated patients with DM and PM [4]. It was evaluated using a 0–4-point scale in one study of patients with IBM [10].

##### Performance of the WB-MRI evaluation system

Six studies examined the performance of a grading system [8,11–15]. Inter-rater reliability for muscle edema grading systems was reported in six studies [8,11–15]. Inter-rater reliability was good to excellent, with kappa coefficients ranging from 0.78 to 1.00, and intraclass coefficients ranging from >0.8 to 1.00 (Table 3). The inter-rater agreement for muscle edema improved in one study when both STIR and DWI sequence scores were acquired [12]. Studies reporting inter-observer agreement for fatty replacement also demonstrated generally good performance. One study reported a kappa coefficient of 0.76, indicating substantial agreement [10], whereas another reported intraclass correlation coefficients ranging from 0.77–0.97, depending on the muscle group analyzed [8]. None of the studies included artificial intelligence or segmentation tools to analyze all or any of these parameters.

#### Utility of WB-MRI in evaluation of IIM

##### Patterns of muscle involvement

Studies have reported that distinctive patterns of muscle or fascia involvement on WB-MRI may discriminate between IIM subtypes [7,9,15,18,19] or between IIM and other myopathic diseases [14]. Among the IIM cohorts, discriminating features included the presence of subcutaneous edema [7] or subcutaneous nodules [9] in DM; widespread muscle increased water signal with truncal involvement in IMNM and ASyS [15]; severe axial and pelvifemoral damage in IMNM [19]; and forearm, thigh, and calf involvement in IBM [15,18]. Another study describing an IBM cohort confirmed a highly distinctive, frequently involved pattern of the medial gastrocnemius, flexor digitorum profunda, and vastus muscles [10]. In comparison, another study reported fascia and/or subcutaneous edema to be common, regardless of the IIM subtype [16], thus diminishing the discriminatory significance of this finding for DM. Six studies detected axial muscle involvement, which would not be evaluated using regional imaging of the proximal limbs [4,9,13,15,16,19]. These studies reported that trunk involvement was commonly detected in 33 %–70 % [4,5,13,15] of patients with IIM, whereas neck muscle involvement was detected in 27 %–60 % [4,13,15,16]. Conversely, one study observed no truncal edema in any patient, despite including newly diagnosed IIM, and concluded that truncal imaging (including paraspinal muscles) could be omitted from WB-MRI protocols without affecting the diagnostic yield [8]. One study observed that the degree of thigh muscle edema was lower in 35 % of patients in the IIM cohort than in other regions [4].

##### Correlation of WB-MRI with clinical variables

Numerous WB-MRI studies have observed a correlation between imaging findings and clinical, pathological, and laboratory parameters, including serum creatine kinase (CK) level [11–13,15], strength of radiologically affected muscle [10], average or global strength [10,13,16], global disease activity [9], and histological features [11] (Supplementary Table 1). However, this was not universal, with some studies reporting no association between the presence and severity of muscle involvement and serum CK levels [10,13,16] or average strength scores [15]. The largest study on WB-MRI evaluation in IIM found that WB-MRI was more frequently abnormal than serum CK levels or electromyography [4], suggesting that this modality may be more sensitive in detecting early/significant muscle disease. This is consistent with other studies that reported that muscle edema was detected in patients with CADM [13] and that MRI-detected edema was present in 46 % of muscles with no clinical weakness [16]. Diffusion-weighted MRI sequences were more sensitive than STIR for the detection of low-grade muscle edema in one study, which was attributed to their higher signal-to-noise background ratio [12].

##### Responsiveness to change in serial WB-MRI studies

One prospective study reported a non-significant reduction in WB-MRI edema scores following nine weeks of intravenous immunoglobulin (IVIg) administration and no improvement in fascial or subcutaneous edema scores [16], raising uncertainty as to whether this modality is useful for monitoring an early treatment response.

### B) DBP-MRI

#### Study characteristics

The mean age of the individuals in the primary articles on DBP-MRI ranged from 20 to 83 years, and the proportion of females ranged from 5.8 % to 100 % (Table 4).

Twenty-three studies evaluated patient populations comprising a mixture of IIM subtypes [20–22,24,25,27–29,31,34,35,38,39,41,42,44,47,49,50,53–55,57], eight studies evaluated only IBM [23,36,50,43,46,52,56,58], seven studies evaluated only IMNM(26,32,33,37,45,48,51), one study evaluated only ASyS [30], a single study evaluated PM/DM without distinguishing between these two subtypes [61], and one study classified patients as IMNM (anti-SRP+), anti-Jo1, and non-Jo1 [54].

The most common subtype of adult IIM in the primary studies was DM (*n* = 699), followed by IMNM (*n* = 535), PM (*n* = 418), ASyS (*n* = 383), IBM (*n* = 357), and CADM (*n* = 51).

Most studies included in this review were observational and cross-sectional [20–31,33,35,36,38–42,46–49,54,55], with either prospective [34,43,52] or retrospective [22,32,37,43–45,50,51,53] design for data collection. One study reported MRI data from a randomized placebo-controlled phase 2b trial [56].

One study examined newly diagnosed IIM [35], and another study reported that 73 % of patients were newly diagnosed with IIM without prior treatment [33]. The remaining studies examined cohorts of patients with longstanding or mixed disease durations. Disease duration, reported in 26 studies, ranged from two months to 22 years [21–23,26–28,30,31,33,34,36–38,41,43,45–49,51–55].

Among these studies, 16 had a control group, the majority of which consisted of other forms of noninflammatory myopathies [22,25–27,30,33–35,38,41,42,45,47,53,57,58], and 16 studies included details of patient treatment regimens [20,21,24,26,28,30,32–35,37,45,47,49,51,56]. Among these, only one study in the entire group was immunosuppression-naïve at the time of MRI acquisition [35].

Of the studies analyzed, 29 focused on assessing the use of DBP-MRI for diagnosing and/or differentiating inflammatory muscle diseases [20–25,27,31,33–36,38,39,51,44,46–49,52,53,57,58]. Additionally, seven studies investigated the effectiveness of DBP-MRI in evaluating treatment responses for these conditions [26,37,40,45,50,51,56], and two studies examined both diagnostic capabilities and treatment response assessment using DBP-MRI [32,55].

#### DBP-MRI protocols

Various DBP-MRI protocols and procedural approaches have been reported in the literature (Table 5). Seventeen studies utilized 1.5T strength scanners [20,22,24,26,27,29,30,32,36–38,44,46,50–52,55], ten utilized a 3T scanner [21,33,35,40,41,43,45,47,56,58], eight studies mixed scanners strength data [25,31,33,48,49,53,54,57], one study used 1.0T or 1.5T [28], and three studies did not describe the field strength used [23,39,42]. Three studies reported the frequent use of gadolinium-based contrast agents and their derivatives [20,38,43].

Imaging protocols evaluated different regions, with the unilateral or bilateral thigh(s) being the region most frequently interrogated in isolation (*n* = 16) or in association with additional regions, such as the upper limb, calves, and/or pelvis (*n* = 15). An isolated assessment of the upper limb/shoulder girdle was not described in any of these studies (Table 5).

Eighteen studies used a combination of T1 and STIR [22,23,37,30,31,33–36,38,42,44,45,49–51,54,55], eight used a combination of T1, T2 fat saturated and STIR sequences [20,25,28,32,37,39,43,47], two studies used a combination of T1 and T2 fat saturated [22,39], two studies used T2 fat saturated and STIR [21,48], and three studies used only T1 or T2 fat saturated sequences [26,46,52]. Two studies used the association of DWI and STIR sequences [29,53], and one study used the association of T1, T2 fat-saturated, and water fat fraction (FF) sequences [40]. Additionally, three studies employed magnetic resonance spectroscopy (MRS) or quantitative sequences, including T2 mapping and Dixon techniques [56–58].

Regarding the imaging planes studied, 18 used only the axial plane [20,22,24,26,29,32,35–38,44–48,52,56,58], 12 used a combination of the axial and coronal planes [21,25,28,30,31,34,41,49,50,53–55], two used only the axial plane [23,43], and six studies did not report the plane(s) used [27,33,39,40,42,51].

#### DBP-MRI evaluation systems

Two studies conducted a semi-quantitative and qualitative analysis [20,53], with all remaining studies applying quantitative, semi-quantitative, or binary scale muscle evaluation systems [21–30–52,54,55,56–58] (Table 6). Thirty-four reported muscle edema [20–37,39,41–43,45–55,58], 14 reported muscle atrophy, muscle volume or cross-sectional area ([20],22,024 [31,34,36,37,41,44,53,54,56],), and 31 reported muscle fatty replacement or fat fraction [20,22–27,30–34,36,37,40,42,43,45–58].

#### Evaluation of muscle edema

Regarding MRI evaluation systems (Table 6), 34 studies evaluated muscle edema with differing patterns of muscle involvement depending on the regions evaluated and type of the IIM. Nineteen studies evaluated muscle edema using semi-quantitative grading scales [20,22,25,26,28–33,36,37,39,42,45,47,49,53,55], whereas nine used binary scoring (present/absent) of individual muscles or regions [23,24,29,34,43,48,49,51,53]. The semi-quantitative grading systems vary from 0–3-point to 0–17-point scales. Six studies used other methods [21,27,31,40,41,46], such as visual analog scores; muscle edema defined as typical, consistent, or atypical patterns; manual annotation using specific software tools; or other quantitative assessments. Five studies [38,40,44,56,57] did not report muscle edema.

Fascial edema, subcutaneous oedema and/or adipose tissue were evaluated in eleven studies [25,29,32,34,36–38,47,49,50,54].

#### Evaluation of fatty muscle replacement

Thirty-one studies reported fatty replacement using either semi-quantitative grading (*n* = 17) [20,22,23,25,26,30,31,33,36,37,42,45,47–49,53] or binary scoring (*n* = 4) [24,25,34,50]. The remaining studies used other methods to report fatty replacement or quantify fat fraction, such as qualitative descriptors, manual analyses, manual drawing using specific software tools or other quantitative systems [27,40,43,46,52,55–58].

#### Evaluation of muscle atrophy

Muscle atrophy, volume or cross-sectional area was assessed in 18 studies [20,22–24,31,34,36–38,40–42,44,45,52–54,56], using semi-quantitative grading scores [22,36,37,42,53], binary scale (present/absent) [20,53,54,34,54], or other methods (measurement of cross-sectional area and muscle volume analysis) [20,31,38,41,42,46,45,50,56]. Muscle volumes or muscle area were evaluated as subjective analyses [20], semi-quantitative [45], or quantitative approaches [40,44,46,52,56].

#### Performance of the DBP-MRI evaluation system

Ten studies examined the performance of reported grading systems [20,28,29,38,42,44,50,51,53,55] (Table 6). Significant variability was observed in the analysis. Two studies reported the inter-reader reliability of muscle edema grading scales using the intraclass correlation coefficient [28,55], whereas two analyzed the inter-rater reliability using kappa [20,42]. Four studies showed interobserver analysis of muscle edema grading scales, fatty replacement, muscle atrophy, subcutaneous adipose tissue, and/or fascia area [29,38,50,53], with coefficients ranging from 0.64 to 0.98. Two studies analyzed intra-observer agreement with coefficients of 0.93 [29] and 0.86 [53]. No studies have used artificial intelligence or segmentation tools to analyze these parameters.

#### Utility of DBP-MRI in evaluation of IIM

##### Patterns of muscle involvement

DBP-MRI offers detailed insights into localized muscle involvement in IIMs, with 25 studies reporting on specific patterns of muscle involvement [20,22,28,30–33,38,41,42,45–54]. Eleven studies reported that DBP-MRI findings differ between IIM subtypes [20,22,24,28,31,34,38,42,49,54,55], whereas eight studies demonstrated differences between IIM and other myopathic conditions [22,27,33,40,44,53,57,58]. One study highlighted differences in DBP-MRI findings between pathologically-confirmed IIM and non-IIM in a cohort of patients with potential presenting IIM symptoms [25], and six studies reported differences between IIM and healthy controls [30,35,41,44,47,52].

Several studies have reported promising results regarding the performance of DBP-MRI in distinguishing IIM from other conditions. One study reported that MRI of the upper legs or upper arms achieved 91 % sensitivity, which was greater than that of muscle biopsy (77 %), but 69 % specificity for distinguishing IIM from non-IIM [25]. Another study [27] defined and validated a characteristic pattern of muscle involvement in IBM as fatty replacement of the distal quadriceps with relative sparing of the rectus femoris, along with other supporting criteria. This pattern distinguished IBM from non-IBM with 95 % accuracy. An additional study [38] identified a characteristic pattern of subcutaneous, fascial, and muscle edema with a peripheral distribution and/or honeycomb pattern in DM, which discriminated DM from other IIM subtypes with 72.2 % sensitivity and 88.5 % specificity. One of the largest DBP-MRI studies reported that the positive predictive value of patterns of muscle involvement of thigh MRI patterns of muscle involvement for distinguishing IIM subtypes was suboptimal; however, the negative predictive value was excellent for IBM (94.7 %), IMNM (93.1 %), and very good for DM (88.3 %) [31].

##### Correlation of DBP-MRI with clinical variables

Nineteen studies examined the relationship between DBP-MRI findings and clinical variables, yielding mixed results [20,21,23,25,26,29,30,33,35,41–44,46,47,50–52,55] (Supplementary Table 2). Nine studies demonstrated a correlation between biomarkers of muscle edema and serum CK levels [21,23,29,30,33,41,42,51,55], whereas four studies showed no significant correlation [20,26,35,50].

The relationship between muscle strength and imaging variables has been inconsistent. Three studies reported a negative correlation between muscle strength and muscle edema [35,47,55], whereas seven studies found no significant association [20,23,26,30,36,42,52]. Six studies observed a negative association between strength and fatty replacement [23,46,47,51,52,55], whereas one study reported no such association [20]. Six studies observed an association between strength and atrophy, muscle volume, or contractile cross-sectional area [36,42,44,47,52,55], whereas one study reported no association between strength and atrophy [20]. One study reported that muscle edema had an improved correlation with objective strength measures when subjects with evidence of muscle fatty replacement were excluded [29]. Another study reported an association between strength and a multivariate fatty replacement and edema score, but not each variable individually [26]. A separate study observed a negative association between strength, combined fatty replacement, and atrophy scores [30].

Associations between muscle edema and validated myositis disease activity scores were observed in two studies [21,29]; however, two other studies reported no such association [26,42]. One study reported an association between fatty replacement and the Myositis Damage Index [42].

Regarding functional outcomes, one study found no association between muscle edema and validated measures of function and mobility [23]. Muscle fatty replacement showed a stronger link with function, with three studies reporting a negative association between muscle fatty replacement and validated measures of function and/or mobility [23,46,52]. One study reported an association between muscle volume and function [52].

##### Responsiveness of DBP-MRI to treatment at a single timepoint

Six studies evaluated associations between treatment and DBP-MRI features, yielding mixed results [26,30,37,39,42,51]. Two studies reported that patients who commenced treatment had reduced edema scores [30,51], notably as higher muscle edema burden was associated with an increased risk of subsequent fatty replacement on follow-up imaging [51]. Conversely, two studies found no statistical difference in MRI parameters between treated and untreated patients [39,49], and another study showed no correlation between cumulative prednisolone doses and imaging parameters [42]. One study observed that patients with marked fatty replacement were proved refractory to therapy [26]. Another group studied a cohort treated under standardized treat-to-target protocols with delayed MRI acquisition, relatively late in the disease process [37]. These cases demonstrated minimal fatty replacement, suggesting that intensive therapy may prevent muscle damage [37].

##### Responsiveness to change in serial BDP-MRI studies

Nine studies examined serial DBP-MRI [21,29,32,42,45,51,52] with follow-up imaging conducted at variable intervals for diverse clinical indications. Three studies specifically compared pre- and post-treatment DBP-MRI [32,51,57]. Three prospective studies undertook imaging performed at standardized time points [52,56,57].

Five studies reported reductions in radiological muscle edema scores over time [21,32,45,51,52]. Conversely, one retrospective study showed no significant difference in the total edema burden across the whole cohort (comprising DM, PM, and IBM patients) [42]. Another study noted that changes in DBP-MRI edema scores upon treatment were not consistently paralleled by changes in serum CK levels, strength, or disease activity visual analog scales [29]. Another study observed persistent histological inflammation despite improvement in radiological muscle edema on serial imaging [21]. Conversely, an IMNM cohort study demonstrated that changes in serum CK levels over time correlated with changes in STIR positivity, suggesting that MRI may be a reliable surrogate for disease activity in this IIM subtype [51].

Five studies (including patients with DM, PM, IBM, and IMNM) noted progressive fatty replacement or fat fraction on serial imaging [42,45,51,52,56], whereas one study examined serial T1-weighted images of four IMNM patients and observed unchanged fatty replacement, albeit over an unspecified follow-up duration [32].

## Discussion

This scoping review highlights the substantial heterogeneity in the WB-MRI and DBP-MRI techniques, protocols, and grading systems currently used to evaluate IIM. Despite increasing reliance on MRI as a diagnostic and monitoring tool in IIM, inconsistencies in study design, imaging protocols, and evaluation systems impede standardization and reproducibility. These challenges hinder cross-study comparisons and integration of MRI into clinical and academic practice. Our findings emphasize the need for a unified approach to MRI in IIM, particularly as our analysis demonstrates MRI to be a valuable tool for detecting muscle inflammation, damage, and atrophy. WB-MRI and DBP-MRI may have the potential to characterize disease patterns that may differentiate IIM from other myopathic conditions and distinguish IIM subtypes. Notably, IBM demonstrated a distinctive and discriminatory imaging phenotype across several studies, although further validation is required. Significant gaps in the evidence base persist, reflecting the rarity of IIM and the heterogeneity of imaging protocols.

A central issue is the lack of standardization in MRI protocols, sequences, anatomical coverage, and grading systems. This problem has been well recognized in the field and was the topic of the 255th European Neuromuscular Centre workshop, which convened international experts to develop consensus-based recommendations for MRI use in IIM [67]. These efforts represent a critical first step toward harmonizing the imaging methodology. Our systematic review empirically reinforces this need: the included studies reported a diverse range of protocols with variations in MRI scanners, field strength, sequence combinations, and image planes.

Among the various imaging sequences, STIR is arguably the standard for evaluating muscle edema, as it is present in active IIM [12]. Indeed, several of the included studies employed STIR sequences only. However, as identified in this review, DWI sequences may be more sensitive for the detection of low-grade muscle edema owing to a reduced signal-to-noise ratio. With normative, age-matched, ethnic data, T1 sequences may also add value, as they permit the detection of fatty replacement and are suited to the quantification and anatomical segmentation of muscle volumes. This is particularly relevant in patients who may present later during the course of the disease. Incorporating T1 sequences can enhance the assessment of cumulative muscle damage, and their routine use may be warranted in many clinical contexts. However, it is important to note that the number of additional sequences performed with multiple coils in situ affects the scan acquisition time and patient tolerance. While STIR-only WB-MRI may be conducted in a relatively brief 15–20 min period, adding DWI or T1/T2FS multiplanar sequences can extend the scan times to 60 min. Balancing the utility of additional sequences with resource demands, costs, image artifacts, and patient tolerance is an important consideration.

Measurement systems for evaluating muscle edema and damage also varied widely among the included studies, with semi-quantitative visual grading scales being favored by many investigators. Binary scoring systems are also commonly employed; these offer simplicity but lack granularity. Several studies have referenced existing grading scales. The most cited WB-MRI muscle edema scale was described by Malattia et al. [59]. For fatty replacement, a range of scales have been used, with many originally developed for neuromuscular disorders, such as that first described by Mercuri et al. [63]. The performance of the semi-quantitative visual grading scales was generally acceptable, although some variability in inter-rater reliability was observed, even among trained observers. Importantly, these visual assessments are usually performed by two trained observers. Achieving the same level of standardization may be challenging in a high-throughput clinical setting, where scans may be reviewed by general clinicians and reported by radiologists with a wider range of experience. A major challenge for the field lies in the development of robust, vendor-agnostic MRI quantification systems that are clinically meaningful, validated, and repeatable with agreed reporting templates. Collaboratively addressing these significant challenges will help solidify the role, efficacy, and renumeration of MRI in clinically differentiating presenting IIM cases, performing multi-site clinical trials, and disease surveillance usage. In this context, the application of artificial intelligence (AI), particularly deep learning algorithms and automated segmentation techniques, represents a promising avenue for future research. These technologies may overcome many of the limitations of visual and semi-quantitative grading systems, including inter-observer variability and subjectivity. AI-driven methods offer the potential for fully automated and reproducible quantification of muscle edema, fatty replacement, and atrophy across large heterogeneous datasets. Although no eligible studies using AI were identified in this review, future research should prioritize the development and validation of these tools to improve the standardization and scalability of MRI-based assessment in IIM, particularly in multi-center trials and real-world clinical monitoring.

Regarding diagnostics, our review suggests that DBP-MRI and WB-MRI are valuable non-invasive tools for evaluating IIM. These modalities contribute to the detection of alternate diagnoses (e.g., denervation and muscular dystrophies) and enable assessment of the extent and phenotype of muscle inflammation, damage, and atrophy in IIM. WB-MRI, with its extended coverage, allows for a comprehensive assessment of patchy, axial, paraspinal, or deep-seated occult muscle inflammation that may not be captured with more regional imaging. This whole-body approach is particularly valuable for assessing muscles that may be challenging to evaluate clinically. The IBM subgroup highlights the diagnostic value of WB-MRI profiling over regional imaging approaches, as the combination of vastus medialis and lateralis, gastrocnemius, and forearm flexor involvement is distinctive in this disease entity [10], and regional imaging cannot capture these highly discriminatory patterns. Nonetheless, we acknowledge that thigh DBP-MRI remains the most widely used modality in current clinical practice, owing to its practicality and availability. Indeed, DBP-MRI involving the thigh may have discriminatory value, with the largest DBP-MRI study demonstrating a very good-to-excellent negative predictive value for IBM, IMNM, and DM. However, with increasing standardization, reduced scan times, and growing accessibility, we anticipate that WB-MRI will become more widely adopted, particularly in research settings and comprehensive disease assessment.

In addition to its role in diagnosis, there is interest in potential MRI biomarkers of disease activity with longitudinal patient tracking to assess treatment response. WB-MRI quantification of edema correlated with markers of disease status in many of the studies included in this review, but such associations were more variable in DBP-MRI studies. Features such as fatty replacement and muscle atrophy on DBP-MRI may correlate more reliably with muscle strength than with localized edema, particularly in the context of intermittent corticosteroids or immunosuppressive therapy, underscoring the multifactorial determinants of muscle dysfunction in IIM. Notably, MRI may also detect subclinical muscle involvement, as demonstrated in CADM, in which prominent edema has been observed in muscles with preserved strength. This underscores the potential sensitivity of MRI for disease detection and monitoring beyond clinical examination. Several studies have shown that MRI changes are dynamic, with demonstrable improvement in muscle edema over time, coupled with progressive evidence of fatty atrophy and damage. However, insufficient prospective studies examining the effects of treatment have been conducted to determine the kinetics and treatment responsiveness of imaging parameters.

Notably, our review has some limitations regarding the assessment of the diagnostic utility of MRI in IIM. Specifically, our study focused on the WB-MRI and DBP-MRI protocols and grading scales, leading to the exclusion of studies that did not provide this information. Consequently, some clinical studies exploring the diagnostic use of MRI in IIM or even its role in enhancing diagnostic yields from muscle biopsies may have been omitted. By prioritizing studies that primarily characterized MR changes, we aimed to ensure a more accurate understanding of the specialist requesting and imaging acquisition in IIM assessments and baseline future efforts in the development of standardized systems.

The existing literature has several limitations. While we identified 49 studies conducted over 24 years, many studies were retrospective with small, highly heterogeneous cohorts in terms of IIM subtypes, disease duration, immunotherapeutic exposure, sampling methods, and the presence of a disease control group. Most WB-MRI studies have focused on DM, PM, and IBM, whereas other important subtypes, such as IMNM and ASyS, have been less frequently studied. Conversely, DBP-MRI studies examined a wider range of IIM subtypes but demonstrated particularly striking heterogeneity in imaging protocols and anatomical regions, with approximately half of these studies being limited to thigh imaging. This is noteworthy, as one WB-MRI study included in this review observed that the thigh region was not the most affected region in > 35 % of patients with IIM, raising concerns that thigh-specific DBP-MRI may underestimate disease burden in some cases. Another limitation is that historical studies may have misclassified IIM subtypes, particularly IMNM and ASyS, as PM, reflecting evolving classification criteria over time. Finally, while some studies have reported muscle atrophy, objective evaluation remains challenging. To our knowledge, no normative data on muscle trophic status are available, and the frequently symmetrical pattern of involvement in non-IBM IIM limits the use of contralateral muscles as internal controls.

A major strength of this review is its rigorous comprehensive methodology, conducted by an international working group utilizing a systematic literature search with strict inclusion and exclusion criteria. Importantly, our group focused on both DBP-MRI and WB-MRI in primary adult studies, offering an analysis of the diverse applications of MRI in IIM.

In summary, MRI is a valuable, non-invasive, radiation-free modality for the diagnosis and evaluation of muscle edema, muscle damage, and atrophy in adult patients with suspected IIM. Its applications include the exclusion of alternate diagnoses, disease monitoring, and treatment evaluation. However, methodological heterogeneity and a lack of standardization, particularly regarding MRI protocols, grading systems, and technical approaches, remain an ongoing issue. Future research should prioritize the development, validation, and guidance of standardized MRI protocols and grading systems, as well as the integration of advanced technologies, including automated segmentation and artificial intelligence, to facilitate assessment and follow-up of patients and potentiate multisite collaborations.

## Supplementary Material

Supplementary tables

Supplementary material associated with this article can be found, in the online version, at doi:10.1016/j.semarthrit.2025.152865.

## Figures and Tables

**Table 1:** Study characteristics of whole-body magnetic resonance imaging in idiopathic inflammatory myopathies.

Reference	Year	Country	Study design	IIM subsets (Number of patients)	Age (yrs)	Disease duration	Current treatment	Female (%)	Characteristics of patients

Cantwell et al. [7]	2005	UK	Prospective	2 DM, 5 PM, 5 IBM	52 (mean)	DM: 4 mo (mean), PM: 6 mo, IBM: 23 mo	None	42	33 % acute weakness, 67 % chronic weakness, 17 % subcutaneous edema, 17 % rash
Filli et al. [8]	2015	Switzerland	Prospective	8 DM, 17 PM, 10 OM, 1 JDM, 27 other myopathies	new diagnosis: 68 (22–81) prior diagnosis: 53 (21–71)	new diagnosis: 1 week (range 0–3) prior diagnosis: 80 weeks (20–720)	86 % IS	79	CK median (range), new diagnosis cohort: 103 U/L (47–365); prior diagnosis cohort: 92 U/L (33–1418) MMT8 median (range), new diagnosis cohort: 64.5 (60–80); previous diagnosis cohort: 59 (46–73)
Elessawy et al. [9]	2016	Egypt	Cross-sectional	5 DM, 4 PM, 5 OM, 1 CAM	25.5 (6–44)	≤3 yrs	-	73	93 % muscle weakness; CK median: 798 U/L (IQR 250–3600); MYOACT VAS (0–10) median muscle: 5 (IQR 3–9); constitutional: 5 (2–7); cutaneous: 2 (0–5); skeletal: 1 (0–6)
Huang et al. [4]	2017	China	Retrospective	99 DM, 30 PM	DM: 50.7 ± 15.4 PM: 38.3 ± 15.3	30.8 ± 47.9 mo, 51 % new diagnosis, 49 % relapsing disease	59 % GC	PM: 63 DM: 69	91 % abnormal muscle biopsy, 50 % abnormal EMG, 73 % raised CPK, 77 % skin features, 27 % ILD
Guimaraes et al. [10]	2017	Brazil	Prospective	12 IBM	59.0 ± 9.1	73.0 ± 27.7 mo	-	50	CK 1.48–8.62 U/L (values multiplied by the normal upper limit: women <170 U/L; men <200 U/L)
Milisenda et al. [11]	2019	Spain	Prospective	16 DM	44.5 ± 3.3	100 % new diagnosis	69 % IS	87.5	100 % weakness, 100 % DM skin lesions; 25 % anti-TIF1γ, 12.5 % anti-Jo-1, 6.3 % anti-NXP2, anti-SAE1, or anti-Mi2
Faruch et al. [12]	2019	Spain	Retrospective	35 DM, 9 PM, 4 IBM, 6 IMNM, 6 OM, 15 controls	53±14	57 % recent onset disease	-	72	CK 582±833U/L
Karino et al. [13]	2020	Japan	Retrospective	24 DM, 17 CADM	48 (IQR 42–60)	100 % new diagnosis	None	65.9	CK 486 U/L (IQR 116–4048), 73 % ILD, 24 % RP-ILD, 41.5 % arthritis, 31.7 % fever, 31.7 % anti-MDA5, 24.4 % ASyS, 7.3 % anti-Mi2; 9.8 % anti-TIF1γ
Landon-Cardinal [19]	2020	France	Retrospective	IMNM 42 IBM 60	IMNM: 48.1 ± 15.8 IBM: 66.3 ± 8.3	IMNM 9.8 ± 8.1 yrs IBM 8.9 ± 5.2 yrs	IMNM mean cumulative prednisone dose 16,959 mg IBM not reported	IMNM 63 % IBM 47 %	IMNM: mean CK level, IU/L 606±580 IBM: mean CK level not reported
Fabry et al. [14]	2022	Brazil	Retrospective case control	5 DM, 13 PM, 3 ASyS, 19 FSHD1	59.3 ± 17	6.0 ± 5.9 yrs	-	47	mRS: 1.2 ± 0.4
Matsuda et al. [15]	2022	Japan	Cross-sectional	2 IBM, 2 IMNM, 4 ASyS, 2 AMAM	63.7 (19–83)	1.1 yrs (range 0.08–4)	GC: 20 %	40	CK 2926±2435 U/L, MRC (0–60) sum score 53.5 ± 4.7, 20 % anti-Jo1, 10 % anti-PL7, 10 % anti-SRP, 10 % anti-HMGCR
Walter et al. [16]	2022	The Netherlands	Prospective longitudinal (pre- and post-IVIG)	9 DM, 4 IMNM, 1 ASyS, 4 NSIIM/OM	55 (IQR 36–58)	0.4 yrs (IQR 0.3–0.5)	Pre-IVIG: None	56	100 % muscle weakness, CK 960 U/L (160–49,710), MMT-13: 214 (185–227)
Cavalcante et al. [17]	2024	Brazil	Cross-sectional	10 IBM, 10 IM-mito	IBM 69.1 ± 7.6	87.2 ± 35.7 mo (IBM)	50 % IS	20	100 % muscle weakness, 20 % cramps, 20 % myalgia, 78.6 % fatigue, 60 % gait impairment, 50 % dysphagia; CK 1980.6 ± 1733.7U/L
Zierer et al. [18]	2024	Germany	Cross-sectional	11 IBM, 11 PM/ASyS/OM,12 IMNM, 7 PM-mito	IBM 58 (40–75) PM/ASyS/OM 65 (33–67) IMNM 72 (48–77) PM-mito 64 (33–67)	IBM 36 (6–180) mo, PM/ASyS/OM 12 (0–72), IMNM 11 (2–180), PM-mito 12 (0–72)	IBM 45 %, PM/ASyS/OM 73 %, IMNM 67 %, PM-mito 43 %	IBM 3/11, PM/ASyS/OM 7/11, IMNM 7/12, PM-mito 7/11	IBM (dysphagia 64 %, myalgia 27 %, MRC SS 57 (43–69); PM/ASyS/OM (dysphagia 18 %, myalgia 64 %, MRC SS 64 (60–70); IMNM (dysphagia 33 %, myalgia 42 %, MRC SS 62 (53–65); PM-mito (dysphagia 0, myalgia 43 %, MRC SS 66 (59–70)

Age is expressed as mean ± standard deviation, or median (minimum – maximum, or interquartile range, IQR).

AMAM: anti-mitochondrial antibody-positive myositis; ASyS, anti-synthetase syndrome; CADM: clinically amyopathic dermatomyositis; CAM: cancer-associated myositis; CK: serum creatine kinase; DM: dermatomyositis; EMG: electromyography; F: female; FSHD1: type 1 fascioscapulo-humeral dystrophy; GC: glucocorticoid; IBM: inclusion body myositis; IIM: idiopathic inflammatory myopathy; IQR: interquartile; ILD: interstitial lung disease; IM-mito: inflammatory myopathy with mitochondrial pathology; IMNM: immune-mediated necrotizing myopathy; IS: immunosuppressive; IVIG: intravenous immunoglobulin; JDM: juvenile dermatomyositis; M: male; mo: month; MMT: manual muscle testing; MRC: Medical Resource Council; MRC SS: Medical Resource Council sum score; MRI: magnetic resonance imaging; MYOACT: Myositis Disease Activity Assessment Visual Analog Scale; NSIIM: non-specific idiopathic inflammatory myopathy; OM: overlap myositis; PM: polymyositis; PM-mito: polymyositis with mitochondrial pathology; RP-ILD: rapidly progressive interstitial lung disease; SD: standard deviation; VAS: visual analogue scale; yrs: years.

**Table 2:** Whole-body muscle magnetic resonance imaging protocol details for studies in the idiopathic inflammatory myopathies.

Reference	Magnet strength, Tesla (T)	Analyzed area	Plane	C+	Sequence	N^◦^ examiners	Acquisition time (min*)	T1 (ms)		T2 (ms)		STIR (ms)	
								TR	TE	TR	TE	TR	TE

Cantwell et al. [7]	1.5	WB	Ax/Co	No	STIR	Two radiologists, blinded	12	NA	NA	NA	NA	4200	60
Filli et al. [8]	3	WB, neck to lower limbs. Trunk omitted for rWBMRI	Ax/Co	Yes	T1/STIR	Two radiologists, blinded	WBMRI: 56 rWBMRI 37	702	8.7	NA	NA	3500–6000	257– 353
Elessawy et al. [9]	1.5	WB, 4–6 contiguous sections	Ax/Co	No	T1/STIR	Two reviewers	15–20	600	15	NA	NA	3000–4000	60
Huang et al. [4]	3	Head to toe; 6 consecutive segments	Ax**/Co	No	T1**/STIR/T2**	Two radiologists, blinded	STIR: 10–12; Total: 15	566	10	3464	70	3996	70
Guimaraes et al. [10]	1.5	WB: bilateral upper and lower limbs	Ax	No	T1/STIR	Two radiologists, blinded	-	600	20	-	-	1400	15
Milisenda et al. [11]	1.5	WB	Ax/Co	No	T1/STIR	Two radiologists, blinded. ImageJ software	-	-	-	NA	NA	-	-
Faruch et al. [12]	1.5	WB	Ax	No	T1/STIR T2/DWI	Two radiologists, blinded	DWI: 24 Total: 50	-	-	-	-	-	-
Karino et al. [13]	1.5	WB: neck to ankles	Ax/Co	No	STIR	Two evaluators, blinded	15–20	NA	NA	NA	NA	4000	70
Landon-Cardinal [19]	3	WB	Ax/Co	No	T1	Two imaging specialists, blinded		Co: 600 (T) 800 (Pr) Ax: 507 (T) 800 (Pr)	Co: 9.1 (T) 9.1 (Pr) Ax: 9.4 (T) Ax: 9.8 (Pr)				
Fabry et al. [14]	1.5	WB: head to lower limbs.	Ax/Co	No	T1/STIR	Two radiologists, blinded	~60	300	10	NA	NA	2965	50
Matsuda et al. [15]	3	WB; neck to legs	Ax/Co	No	T1/STIR	Two observers, one blinded	20–35	450	8.7	NA	NA	3000	69
Walter et al. [16]	3	WB	Ax/Co	No	T2	One radiologist, blinded	-	NA	NA	-	-	NA	NA
Cavalcante et al. [17]	1.5	WB	Ax	No	STIR/FSE	Two radiologists, blinded	60	-	-	-	-	450–600	minimum
Zierer et al. [18]	3	WB	Ax	No	T1/FSE	One radiologist, one evaluator	-	-	-	-	-	-	-

Ax: axial; *C*+: contrast; Co: coronal; DWI: diffusion weighted imaging; FSE: fast spin echo; min: minute; ms: millisecond; NA: not applicable; Pr: prisma; rWBMRI: restricted protocol WBMRI; STIR: short tau inversion recovery; T: trio; TE: time to echo; TR: repetition time; WB: whole-body; WBMRI: whole-body magnetic resonance imaging.

* rounded to the nearest full minute;.

** thigh region only.

**Table 3:** Muscle whole-body magnetic resonance imaging grading system in idiopathic inflammatory myopathies.

Reference	Abnormality	Muscle evaluation system	Assessed area	Performance of grading system

Cantwell et al. [7]	MO, FO	Qualitative	WB - no details	-
Filli et al. [8]	MO, FR	SQS: MO (0–2-point scale) and FR (0–4-point scale)	Deltoid, RC, Bi, Tri, Quad, Abd, GM, Gm, hamstring, TA, Pectorales, trapezius, SA, intercostals, AW, LA, EST, ESL, iliopsoas	IR: MO: ICC 0.89–1.0; FR: ICC 0.77–0.97
Elessawy et al. [9]	MO, FO, SCO	SQS: MO (0–3-point scale). Binary scale: FO and SCO	Neck, upper limb, thorax, abdomen, pelvic girdle, thighs, calf region	-
Huang et al. [4]	MO, FR, MA, SCO	Qualitative. Binary scale	Neck, upper extremity, chest, lumbar muscle, pelvis, lower extremity	-
Guimaraes et al. [10]	MO, FR, MA	SQS FR (0–4-point scale), MO (0–2-point scale), MA (0–3-point scale)	64 muscle groups per patient; bilateral upper and lower limbs	IR: MO: *k* = 0.85; FR *k* = 0.76; MA=0.65
Milisenda et al. [11]	MO, FR, FO	Quantitative analysis of MO using ImageJ SQS: MO and FO (0–3-point scale). Binary scale: FR	WB - Quantitative: 1 muscle. Semi-quantitative: body was divided into 4 anatomical regions and evaluated	IR: ICC > 0.8
Faruch et al. [12]	MO, FR	Quantitative analysis using ADC values SQS: MO and FR (0–3-point scale)	WB - Quantitative: 1 region (the largest cross-sectional area in the axial plane). Semi-quantitative: 78 separate muscles were evaluated	IR: STIR: *k* = 0.84; DWI and STIR: *k* = 0.97
Karino et al. [13]	MO, FO	Binary scale: MO and FO	42 muscle groups; symmetrical muscles scored separately. All muscles were evaluated on both axial and coronal sections	IR: *k* = 0.78–1.00
Landon-Cardinal [19]	FR	SQS: FR (0–4-point scale)	55 bilateral muscles across axial, shoulder, pelvic, lumbar, and lower limb regions	-
Fabry et al. [14]	MO, FR	Ability of radiologists and DLT to discriminate IIM versus non-IIM	Shoulder, thigh, calf	Correct classification: Reader 1: 95 %; Reader 2: 87.5 %; DLT: 77 %
Matsuda et al. [15]	MO, FO	SQS: MO (0–3-point scale). Binary scale: FO	54 muscles selected (total 108 bilaterally) in the following regions: neck (4 pairs of muscles), upper arm (4 pairs), thoracic trunk (9 pairs), abdominal trunk (6 pairs), pelvis (10 pairs), thigh (14 pairs), calf (7 pairs)	IR: *k* = 0.84
Walter et al. [16]	MO, FR, FO	SQS: MO (0–2-point scale). Binary scale: FR, FO	Semi-quantitatively scored in 36 muscle groups: cervical, deltoid, supraspinatus, infraspinatus, Bi, Tri, forearm flexors and extensors, Gluteal, iliopsoas, Sa, hip adductors, Quad, hamstring, TSL, TA, peroneus, and Ga	-
Cavalcante et al. [17]	MO, FR	SQS: FR (0–4-point scale). Binary scale: MO	7 regions (right and left: arms and forearms; pelvis, thighs, legs	-
Zierer et al. [18]	MO, FR	SQS: FR (0–5-point scale). Binary scale: MO	Head/neck (6 muscles), shoulder/thorax (14 muscles), trunk/pelvis (12 muscles), upper leg (15 muscles), lower leg (10 muscles)	-

Ab: abductor brevis; AB; abdominal wall; ADLL: anterior distal lower limb; AL: adductor longus; AM: adductor magnus; Bi: biceps; BF: biceps femoris long head; BR: brachioradialis; CI: confidence interval; DL: digitorum longus; DLT: deep learning tool; ECR: extensor carpi radialis; ECU: extensor carpi ulnaris; EDC: extensor digitorum communis; EDL: extensor digitorum longus; ESL: erector spinae lumbar; EST: erector spinae thoracic; FCU: flexor carpi ulnaris; FCR: flexor carpi radialis; FO: fascial edema; FR: fatty replacement; HIS: high signal intensity; Ga: gastrocnemius; Gl: gluteus; GM: gluteus maximus; Gm: gluteus medius; Gr: gracilis; HL: hallucis longus; ICC: intraclass correlation coefficient; IIM: idiopathic inflammatory myopathy; InB: intraobserver agreement; IoB; interobserver agreement; IR: inter-rater reability; QF: quadratus femoris; Quad: quadriceps; LA: latissimus dorsi; MA: muscle atrophy; MO: muscle edema; MV: muscle volume; OE: obturator externus; OI: obturator internus; PB: peroneus brevis; PL: peroneus longus; PT: pronator teres; RF: rectus femoris; PDLL: posterior distal lower limb; Pe: pectineus; RC: rotator cuff; SA: serratus anterior; Sa: sartorius; SC: subcutaneous; SCO: subcutaneous edema; So: soleus; SQS: semi-quantitative scoring; SM: semimembranosus; ST: semitendinosus; STe: supinator teres; STIR: short tau inversion recovery; TA: tibialis anterior; Th: thighs; TP: tibialis posterior; Tri: triceps; TSL: tensor fasciae latae; VAS: visual analogue score; VI: vastus intermedius; VL: vastus lateralis; VM: vastus medialis.

**Table 4:** Studies in dedicated body-part magnetic resonance imaging in myositis: general data from the patients with idiopathic inflammatory myopathies and control groups.

Reference	Year	Country	Study design	IIM subsets	Age (yrs)	Disease duration	Current treatment	F (%) or F/M ratio	Characteristics of patients

Dion et al. [20]	2002	France	Cross-sectional	25 PM, 25 IBM	IBM 60.8 ± 13.0, PM 49.1 ± 12.5	-	GC: 24 PM, 20 IBM; MTX: 9 PM, 3 IBM; IVIG: 8 PM, 15 IBM; AZA: 3 PM, 2 IBM	IBM (9F/17 M)	Muscle strength mean score IBM 63.0 ± 8.3, PM 5.0 ± 11.6; 1 patient with anti-Jo1(+); IBM CK 584 ±182 U/L, PM 3329±1238U/L
Tomasová Studýnková et al. [21]	2007	Czech Republic	Cross-sectional	20 DM, 9 PM	53.3 (24–78)	2.3 yrs (2 mo-11 yrs)	GC (*n*=¼17), 5 MTX, 3 CsA, 1 AZA	23F/6M	Initial CK: range 0.35–120 (upper normal limit of 2.84ukat/L); Global clinical activity: range 0.2–5.5 (reference 0–10), VAS-M 0.1–4.2 (reference 0–10)
Degardin et al. [22]	2010	France	Cross-sectional / retrospective	1 DM, 4 PM, 4 IBM	DM 65, PM 64.3 ± 8.4, IBM 63.5 ± 5.3	DM 0.6 PM 5.6 ± 7.7 IBM 6.8 ± 7.7	-	-	-
Cox et al. [23]	2011	The Netherlands	Cross-sectional	32 IBM	68±9	12±5 yrs	-	13	The most affected muscles: the ventrally located muscles in the arm, the upper, and the lower leg. CK elevated in 88 % of cases. CK median 739 U/L (121–3360) for men, and 265 U/L (44–802) for women
Miranda et al. [24]	2014	Brazil	Cross-sectional	11 DM, 11 PM	DM 50.9 PM 49.9	-	GC: DM 3,0 g, PM 1,4 g/; IS: 3 DM, 6 PM	DM 9F, PM 8F	DM CK 1340 U/L (158–3489), PM CK 870 U/L (207–2519); Both with significant weakness of four proximal limbs
Van De Vlekkert et al. [25]	2015	The Netherlands	Cross-sectional	7 DM, 1 IMNM	50±14	-	-	31F/17M	Muscle features are described individually; CK 37–847 U/L; 3 anti-MDA5, 2 anti-ARS, 6 anti-TIF1γ
Zheng et al. [26]	2015	The Netherlands	Cross-sectional	12 IMNM	39.9 ± 12.7	22.8 ± 20.6 mo	12 GC; At least one: MTX, AZA, CsA, Tacro, CYC	6F/6M	CK 5200±3314 U/L (range 2410–13,265); 12 anti-SRP; proximal and symmetrical limb muscle weakness in all patients; MYOACT score of 15.1 ± 6.3
Tasca et al. [27]	2015	Italy	Cross-sectional	8 DM, 22 PM, 19 IBM	67 (46–85)	Range: 41–78 yrs (age at onset)	-	5F/14M	Described individually (distal upper limb weakness; proximal and distal lower limb weakness): from mild to severe. Facial weakness and dysphagia: presence or absence
Pipitone et al. [28]	2016	Italy	Cross-sectional	31 DM, 40 PM	DM 20±31 PM 53±69	DM 20±31 mo, PM 53±69 mo	GC: 23 DM, 28 PM; IS: 17 DM, 25 PM	DM (23F/8 M) PM (31F/9 M)	MRC≤4 in at least four proximal muscle groups were considered in any case a prerequisite for active myositis: 84 % DM, 90 % PM
Barsotti et al. [29]	2016	Italy	Cross-sectional	22 DM 22 PM	53.7 ± 13.2 (24–80)	-	-	38F/13M	CPK 1907 U/L (609–9979) - increased in 35/51. PGA-VAS mean of 4.8 ± 3.2 cm; 37 had clinically active disease; MMT8 was performed in 30 patients, with a mean score of 54±14.
Andersson et al. [30]	2017	Norway	Cross-sectional	68 ASyS	47±13.8 (at diagnosis)	54 ASyS 76 mo (6–232)	GC: (*n* = 66/68); 48 GC + at least one drug; 10 GC as monotherapy Disease course: CYC, AZA, or MTX; *n* = 24/68 had received at least 1 cycle RTX. 2 IS naïve. Maintenance: 27 AZA, 14 MMF, 5 MTX, 3 CsA, 1 Tacro, 1 RTX	45F/23M	CPK median 95 U/L (range 24–1344); MMT14 score 139 (IQR 133–140), MMT4 score 40 (IQR 36–40), FI2 score 211 (IQR 166–301); 53 anti-Jo1, 6 anti-PL7, 9 anti-PL12
Pinal-Fernandez et al. [31]	2017	USA	Cross-sectional	219 DM, 17 ADM 176 PM, 153 IMNM, 101 ASyS	50.4 ± 15.0 (at disease onset)	5.2 ± 6.3 yrs	Patients with anti-SRP were more commonly under IS treatment than those with anti-HMGCR	DM F76 % ADM F82 % PM F66 % IMNM F65 % ASyS F38 %	50 anti-HMGCR, 22 anti-SRP
Villa et al. [32]	2018	Italy	Retrospective	5 IMNM	59–76	-	All patients started GC for at least 6 mo (MRI scanning was performed before therapy in 4 patients) 3 AZA, 4 IVIG, 3 MTX, 2 MMF	2F/3M	CK 5900–9584 U/L; EMG(+) in all; 5 anti-HMGCR
Zhao et al. [33]	2018	China	Cross-sectional	30 IMNM	47.0 (27.0 ± 54.3)	5.0 mo (2.0–24.0)	8 GC for 16.0 (8.5, 27.0) mo; 22 with IMNM had not received GC	17F/13M	80 % neck flexor weakness; 15.6 % myalgia; CK 4618 U/L (3125–7303); 25 anti-SRP
De Lorenzo et al. [34]	2018	USA	Prospective	41 anti-PM/Scl 178 DM 132 ASyS 135 IMNM	Anti-PM/Scl 42.2 ± 15.0, DM 47.1 ± 15.6, ASyS 45.0 ± 13.3, IMNM 51.5 ± 14.9 (at disease onset)	Anti-PM/Scl 6.5 ± 4.7 DM 4.3 ± 3.5 ASyS 4.7 ± 3.9 IMNM 4.0 ± 3.9	GC: 88 % Anti-PM/Scl, 83 % DM, 96 % ASyS, 75 % IMNM AZA/MTX/MMF/IGIV/RTX: 41 % anti-PM/Scl, 26 % DM, 58 % ASyS, 27 % IMNM	DM F35 ASyS F73 % IMNM 63F	Muscle weakness: anti-PM/Scl (93 %), DM (85 %), ASyS (65 %), IMNM (96 %) Anti-PM/Scl CK 138 U/L (IQR 80–472), DM 117 U/L (68–290), ASyS 282 U/L (114–963), IMNM 1401 U/L (2000–8990)
Ran et al. [35]	2018	China	Case-control	42 DM/PM	41 (14–73)	-	No treatment before MRI	26F/16M	31 clinically symmetric muscle weakness had MMT scores. Elevated CK in 26 of 31 DM/PM
Dahlbom et al. [36]	2019	Sweden	Cross-sectional	19 IBM	65.5	7.1 (range 3–22) yrs	-	F10.5 %	CK median at time of diagnosis: 10.2 ukat/L (3.4–1.9) (normal < 3.5 ukat/L)
de Souza et al. [37]	2019	Brazil	Retrospective	13 IMNM	53.5 ± 14.2 (at diagnosis)	4.0 mo	9 GC (induction); 2 current GC, 11 IVIG, 4 MTX, 2 RTX, 1 MMF, 5 AZA, 1 CsA	9F/4M	MMT8 68, HAQ 1.80, patients’ VAS median 7.0, physicians’ VAS median 6.0, MYOACT median score 2.6; 4 anti-HMGCR, 9 anti-SRP
Ukichi et al. [38]	2019	Japan	Cross-sectional	36 DM 17 ADM 19 PM	DM 53.5 ± 14.3 ADM 50.5 ± 11.7 PM 64.1 ± 11.4	DM 157.9 ADM 205.4 PM 495.6 (days)	Not taking any medications: 16 DM, 16 ADM, 15 PM. The remaining patients were under treatment with GC, AZA, CsA, or MTX.	DM F66.7 % ADM F47.1 % PM 63.2 %	Muscle weakness: DM 94.4 %, ADM 11.8 %, PM 79.0 %; CK: DM 1771.9 ± 2257.8 U/L, ADM 384.6 ± 586.9 U/L, PM 1840.1 ± 1651.4U/L
Aoki et al. [39]	2019	Japan	Cross-sectional	20 DM, 2 PM, 4 ADM, 1 IBM, 5 IMNM, 12 ASyS	60 (range 31–85) at disease onset	-	9 GC, 0 IS	F56 %	Median MRC sum score: 52; median max CPK 640 U/L (IQR322–2599); 6 anti-Jo1, 3 anti-PL12, 1 anti-EJ, 1 anti-KS, 9 anti-MDA5
Marty et al. [40]	2019	France	Case-control	7 IBM	69.1 ± 9.9	-	-	7M/0F	-
Wang et al. [41]	2019	China	Case-control	7 DM, 13 PM	49 (29–69)	32.3 mo (range, 2–144)	-	1F/1.2M	CK 1816 U/L (range 15–4055)
Day et al. [42]	2019	Australia	Cross-sectional	28 DM/PM, 12 IBM, 18 IMNM	61 (47–75)	-	-	F55 %	MMT8 IMNM 70 (64–78), DM/PM 76 (65–80), IBM 63 (59–74) CK IMNM 4145 U/L (2200–11,028), DM/PM 1352 U/L (566–3996), IBM 637 U/L (403–955) 2 anti-Mi2, 1 anti-Jo1, 4 anti-PL7, 1 anti-HMGCR, 1 anti-SRP
Lassche et al. [43]	2019	The Netherlands	Retrospective and prospective	8 IBM	58.5 ± 1.8	6.4 ± 2.5	-	1F/7M	MRC-score: quadriceps 4.0 ± 0.9 and tibialis anterior 4.5 ± 0.8, CK 772.3 ± 261.1U/L
Müller et al. [44]	2020	Germany	Retrospective	1 PM, 3 IBM, 2 IMNM, 18 myopathies, 13 neuropathies	55.0 ± 11.7	-	-	50	CK 473±261.6U/L
Zhao et al. [45]	2020	China	Retrospective	48 IMNM	47.0 (IQR 27.0–54.3)	5.0 (IQR 2.0–24.0)	95.8 % GC; 54.2 % IVIG, 43.8 % MTX, 6.3 % AZA, 6.3 % Tacro	34F/14M	CPK 4618.0 U/L (IQR 3124.8–7303.0); EMG (+) 90 %; 22/46 (47.8 %) ANA, 28/48 (58.3 %) isolated anti-SRP, 12/48 (31.3 %) anti-SRP+Ro52, 3/48 (6.3 %) anti-SRP+PM/Scl75, 1/48 (2.1 %) anti-SRP+Jo1, 1/48 (2.1 %) anti-SRP+PL12
Ansari et al. [46]	2020	France	Cross-sectional	16 IBM	70.1 (61.6–78.6)	12 yrs	-	F50 %	Mean MRCSLL scores: 34.8 ± 11.3; mean IBMFRS score 30.3 ± 7.3
Araujo et al. [58]	2020	France	Cross-sectional	114 IBM 30 DMD 55 Controls	Median (IR) 64.0 (12.4) Whole range 35.8–84.9	-	-	IBM 54/60	-
Farrow et al. [47]	2021	UK	Cross-sectional / Case-control	6 DM, 10 PM	50 (24–76)	5 yrs (range 1 mo - 22 yrs)	9 GC, 16 IS	F62.5 %	CK median 1000 U/L (range 70–12,802); 5 anti-Jo 1, 2 anti-PM/Scl75, 2 anti-PM/Scl100, 1 anti-PL12, 5 anti-Ro52, 1 anti-La, 3 anti-Sm/RNP, 3 antichromatin, 1 ACA
Lee et al. [48]	2021	China	Cross-sectional	6 IMNM	-	6 (IQR 3–12) mo	-	F 83 %	CK 2603–10,537 U/L; 6 anti-HMGCR
Reyngoudt et al. [57]	2021	France USA	Prospective	21 IMNM 21 IBM 10 GNEM 19 Duchenne muscular dystrophy 12 DYSF 7 LGMD2I 7 Pompe disease 5 SMA	IMNM 49.1 (35.2–63.7) IBM 66.7 (58.8–71.2)	Age at onset IMNM 46.6 (32.8–50.2) yrs IBM 63.0 (53.1–67.5) yrs	-	IMNM 10/11 IBM 10/11	% of ambulant subjects at year 1 100 % IMNM and IBM
Zhang et al. [49]	2022	China	Cross-sectional	56 DM, 61 IMNM, 51 ASyS	ASyS 52.3 (34.2–58.9) DM 43.1 (13.1–61.8) IMNM 46.5 (27.8–55.9)	ASyS 0.56 yrs; DM 0.42; IMNM 1.03	41.7 % GC, 41.7 % IS	ASyS F64 % DM F69 % IMNM F60 %	ASyS: muscle weakness 56.9 %, median CPK 345 U/L (97–3546); 24 anti-Jo1, 11 anti-EJ, 9 anti-PL7, 3 anti-OJ, 2 anti-PL12, 2 anti-Zo
Oto et al. [50]	2023	Japan	Retrospective	15 DM, 13 CADM	55 (42–63)	-	Triple therapy: DM 60.0 %, ADM 84.6 %	F 79 %	Myalgia DM 40 % and ADM 53.8 %; median CK DM 240 U/L (IQR 16.5–475) and ADM 134 U/L (41–216)
Laurent et al. [52]	2022	UK	Prospective	30 IBM	65.6 ± 10.1	9 yrs	-	F16.7 %	Mean 6MWD 325±153 m; dysphagia 53.3 %, sIFA in arbitrary units 47.6 ± 23.8
Fionda et al. [51]	2023	Italy & Spain	Retrospective	22 IMNM	59.5 ± 17.4	Median: 4 (range 0–330) mo	-	F 71 %	MRC-baseline: 26–60; CK 205–30000U/L 7 anti-HMGCR, 8 anti-SRP, 7 seronegative
Barsotti et al. [53]	2023	Italy	Retrospective	30 DM, 59 PM, 2 IBM	58.8 ± 13.2	2.3 yrs	-	F70.3 %	Muscle pain 37.3 %, functional status (Walton Gardner-Medwin scale) 3.1 ± 2.1
Kimura et al. [54]	2024	Japan	Cross-sectional	5 anti-SRP 11 anti-Jo1 22 non-Jo1	39 (IQR 20–50); 44 (35–63), 56.5 (44.8–67.3)	3 (IQR 1–4) mo, 2 (1.5–3), 3 (2–0.5.8)	-	Anti-SRP F80 %, anti-Jo1 F54.5 %, non-Jo1 F86.4 %	MMT iliopsoas; anti-SRP 4 (IQR 4–5), anti-Jo1 4 (4–5), non-Jo1 4 (4–50); quadriceps anti-SRP 4 (4–5), anti-jo1 4 (4.25–5), non-Jo1 4.5 (4–5); hamstring anti-SRP 4.5 (4–5), anti-Jo1 5 (4–5), non-Jo1 4 (4–5); CK anti-SRP 4315 U/L (IQR 2597–9668), anti-Jo1 1800 U/L (1038–1748), non-Jo1 699.5 U/L (171–2150)
Gorijavolu et al. [55]	2024	India	Cross-sectional	32 DM, 5 PM, 19 ASyS, 3 IMNM	38 (30–47)	4 (2–12) mo	-	44F, 15M	Median MMT8 60 (IQR 48–70), CK 1322 U/L (IQR 125–3695.5); anti-Mi2 (27.6 %), anti-Jo1 (22 %), anti-PL12 (12.1 %), anti-Ro52 (17.2 %)
Reyngoudt et al. [56]	2024	France	Prospective	44 IBM	22 IBM placebo 66.7 [58.4–73.8] 22 IBM sirolimus 68.1 [62.8–73.9]	22 IBM placebo 2.8 [1.1–5.6] yrs 22 IBM sirolimus 1.9 [0.5–4.4] yrs	22 placebo 22 sirolimus	22 IBM placebo 12F/10M 22 IBM sirolimus 8F/14M	22 IBM placebo: CK 446.0 [276.3–777.3] / 6MWD (m) 324.0 [264.5–417.3] / Knee flexion strength (Nm) 28.8 [19.8–43.1] / Knee extension strength (Nm) 22.9 [10.8–46.1] 22 IBM sirolimus: CK 314.5 [217.5–652.5] / 6MWD (m) 324.0 392.5 [354.0–552.5] / Knee flexion strength (Nm) 33.7 [23.2–46.6] / Knee extension strength (Nm) 22.1 [15.1–41.9]

Age is expressed as mean ± standard deviation, or median (minimum - maximum, or interquartile range, IQR).

ADM: amyopathic dermatomyositis; ARS: anti-aminoacyl-tRNA synthetase; ASyS: anti-synthetase syndrome; AZA: azathioprine; CK: serum creatine phosphokinase; CsA: cyclosporine; CYC: cyclophosphamide; DM: dermatomyositis; F: female; GC: glucocorticoid; HMGCR; 3-hydroxy-methyl-glutaryl coenzyme reductase; IBM: inclusion body myositis; IBMFRS: inclusion body myositis functional rating scale; sIFA: sIBM Physical functioning assessment; IIM: idiopathic inflammatory myopathy; IMNM: immune-mediated necrotizing myopathies; IQR: interquartile; IS: immunosuppressive; IVIG: intravenous immunoglobulin; MMF: mycophenolate mofetil; mo: month; MRC: Medical Resource Council; MRCSLL: Medical Research Council score of the lower limbs; MRI: magnetic resonance imaging; MTX: methotrexate; MYOACT: Myositis Disease Activity Assessment

Visual Analog Scale; 6MWD: 6-minute walk distance; PGA: physician global assessment; PM: polymyositis; RTX: rituximab; SRP; Tacro: tacrolimus; VAS: visual analogue score; VAS-M: visual-analogue score - medical; yrs: years.

**Table 5:** General characteristics of dedicated body-part magnetic resonance imaging in myositis from available studies in patients with idiopathic inflammatory myopathies.

Authors [ref]	Magnet strength Tesla (T)	Analyzed area	Plane	C+	Sequences	N^◦^ examiners	Acquisition time (min*)	T1 (ms)		T2 (ms)		STIR (ms)		
								TR	TE	TR	TE	TR	TE

Dion et al. [20]	1.5	Th	Ax	Yes	T1W/T2W (STIR or fast T2 fat sat)	Four radiologists, blinded	-	440	11	3400	102	5500	34
Tomasová Studýnková et al. [21]	3.0	Th	Ax/Co	No	T2W/STIR	Two evaluators, blinded	-	-	-	-	-	-	-
Degardin et al. [22]	1.5	Right shoulder, right A, P, upper and lower L	Ax	No	T1/STIR	Two radiologists, blinded	-	-	-	-	-	-	-
Cox et al. [23]	-	31 P and H, 9 S, 14 L	Tr	No	T1/STIR	Two radiologists, blinded	-	600	20	-	-	1400	15
Miranda et al. [24]	1.5	Th	Ax	No	T1/T2	Two radiologists, blinded	-	-	-	-	-	-	-
Van De Vlekkert et al. [25]	1.5 or 3.0	Upper A or upper L	Ax/Co	No	T1W/T2W/STIR	One radiologist	-	420	14	4000	75	4000	69
Zheng et al. [26]	1.5	Th	Ax	No	T1	Two evaluators, blinded	-	450	12	-	-	5000	90
Tasca et al. [27]	1.5	Lumbar spine to ankles	-	No	T1W/STIR	Three neurologists, blinded	-	-	-	-	-	-	-
Pipitone et al. [28]	1.0, 1.5 or 1.5	Th	Ax/Co	No	T1/T2/PD/STIR	One radiologist	-	-	-	-	-	2500, shortest, shortest	55, 60, 60
Barsotti et al. [29]	1.5	Th	Ax	No	STIR/DWI	Two evaluators, blinded	3	-	-	-	-	43	3000–4000
Andersson et al. [30]	1.5	Th	Ax/Co	No	T1W/STIR	Two radiologists, blinded	-						
Pinal-Fernandez et al. [31]	1.5 or 3.0	Th	Ax/Co	No	T1/STIR	Two radiologists, blinded	-	680–790	8–12	-	-	3500–6800	42–58
Villa et al. [32]	1.5	Upper and lower Li	Ax	No	TSE sequences T1/Fat sensitive, STIR/T2	NR	-	-	-	-	-	-	-
Zhao et al. [33]	3.0	Th, P	-	No	T1W/STIR	-	-	-	-	-	-	-	-
De Lorenzo et al. [34]	1.5 or 3.0	Th	Ax/Co		T1W/STIR	Two radiologists, blinded	-	680–790	8–12	-	-	3500–6800	42–58
Ran et al. [35]	3.0	Th	Ax	No	T1W/T2W	Two radiologists, blinded	20	720	7.2	3600	85.5	-	-
Dahlbom et al. [36]	1.5	Th, L, A	Ax	-	T1W/STIR	One radiologist	-	-	-	-	-	-	-
de Souza et al. [37]	1.5	Th	Ax	No	T1W/T2W/FATSAT/STIR	Two rheumatologists, blinded	-	-	-	-	-	-	-
Ukichi et al. [38]	1.5	Upper A and/or Th	Ax	Yes	STIR (*n* = 88) / Gd-T1WI (*n* = 79)	One radiologist one rheumatologist	GdT1WI: 2.68 SITR: 2.55	530	11	-	-	4000	25
Aoki et al. [39]	-	Th, A	-	No	T1W/T2W/STIR	-	-	-	-	-	-	-	-
Marty et al. [40]	3.0	Th		No	T1/T2/H20/FF	-	0.83	10,000	5.08	-	-	-	-
Wang et al. [41]	3.0	Th	Ax/Co	No	T1/T2W T2 fat sat	Two radiologists, blinded	T1 Co 5min17s T2 Co 3min23s T2 Ax 2min41s	6.73	2.46/3.69	3050–5330	119	-	-
Day et al. [42]	-	Th, P, Li	-	-	T1W/STIR	Two evaluators, blinded	-	-	-	-	-	-	-
Lassche et al. [43]	3.0	Upper and lower L	Tr	No	T1/T2/STIR	-	-	600	13	3000	77–123.2	4100	41
Müller et al. [44]	1.5	4 stations: neck/chest, abdomen/pelvis, upper and lower legs	Ax	No	T1/STIR	Two radiologists, blinded. Deep learning tool	-	902	17	3971	64	NA	NA
Zhao et al. [45]	3.0	Th	Ax	No	T1/STIR	-	-	-	-	-	-	-	-
Ansari et al. [46]	1.5	Th, lower L	Ax	No	T1W	Two experts, blinded	-	578	11	-	-	-	-
Araujo et al. [58]	3 T	Th / L	Axial	No	T2, single voxel 1H-MRS	-	-	-	-	3000	9.5		-
Farrow et al. [47]	3.0	Th, L	Ax	No	T1/T2/STIR	Two evaluators, blinded	T1 2min19s T2 2min05s STIR 3min18s	697	9.1	1500	9.6, 9.4, 153.6	6550	87
Lee et al. [48]	1.5 or 3.0	Th, P	Ax	No	T2/STIR	One neurologist, one radiologist	5–6	500–700	7–10	6000–7500	60–75	-	-
Reyngoudt et al. [57]	3 T France 1.5T USA (only patients with DMD)	Th / L	Axial	No	3D gradient echo (3-point Dixon)	-	9 min per segment (thigh or lower leg)	10	-	-	-	-	-
Zhang et al. [49]	1.5 or 3.0	Th, H	Ax/Co	No	T1/STIR	Two physicians, blinded	-	-	-	-	-	-	-
Oto et al. [50]	1.5	Bilateral Th and upper Li	Ax/Co	Yes	T1/STIR	One radiologist, two rheumatologists, blinded	GdT1WI: 2.68 SITR: 2.55	530	11	-	-	4000	25
Laurent et al. [52]	1.5	Th	Ax	No	T2	Two evaluators, blinded	-	-	-	-	-	-	-
Fionda et al. [51]	1.5	P, lower L, scapular girdle, A	-	No	T1/STIR	Two neurologists, blinded	-	-	-	-	-	-	-
Barsotti et al. [53]	1.5 or 3.0	Th, P	Ax/Co	No	STIR/DWI/DUOL ECHO	Two radiologists, blinded	20–30	-	-	-	-	3300–3900	50–70
Kimura et al. [54]	1.5 or 3.0	Th	Ax/Co	No	T1/STIR	-	-	-	-	-	-	-	-
Gorijavolu et al. [55]	1.5	Th, P	Ax/Co	No	T1/STIR	Two evaluators, blinded	-	-	-	-	-	-	-
Reyngoudt et al. [56]	3 T	One-third of the femur (distally) and at the widest part of the calf (both sides)	-	No	Quantitative water-fat / T2; 31P MRS	-	50 min	10	2.75 _ 3.95 _ 5.15	3000	9.5–161.5	-	-

A: arm; Ax: axial; *C*+: contrast; Co: coronal; DWI: diffusion weighted imaging; H: hip; L: leg; Li: limb; min: minute; ms: millisecond; MRI: magnetic resonance imaging: shoulder; NR: not reported; PD: proton density weighted; ROI: region of interest; Sa; sagittal; STIR: short-tau inversion recovery; TE: time to echo; Th: thigh; Tr: transversal; TR: repetition time; P: pelvis; VAS: visual analogue score.

* rounded to the nearest full minute.

**Table 6:** General data of dedicated muscle body-part magnetic resonance imaging from available studies in patients with idiopathic inflammatory myopathies.

Reference	Abnormality	Muscle evaluation system	Assessed area	Performance of grading system

Dion et al. [20]	MO, FR, MV, MA	SQS: MO and FR (0–4-point scale); Subjective analysis: MV Binary scale: location of abnormalities and MA	RF, VI, VL, VM, BF, BL, ST, SM, AL, AM, Gr, Sa	IR agreement: Cohen’s k on 46 patients (PM, DM, or IBM); k of each sequence: SE T1 FR (*k* = 0.93), T2 fat saturation (*k* = 0.90), STIR (*k* = 0.89), SE T1 postcontrast fat saturation (*k* = 0.90)
Tomasová Studýnková et al. [21]	MO	Score by VAS (0–10 cm): extent, intensity and total area affected	Bilateral thighs	-
Degardin et al. [22]	FR, MO, MA	SQS: FR, MO, and MA (0–3-point scale)	Lower and upper limbs	-
Cox et al. [23]	MO, FR, MA	SQS: FR (0–3-point scale). Binary scale: MA and MO	Shoulder, region, upper arm, forearm, Pe, upper leg, lower leg; total of 68 separate muscles	-
Miranda et al. [24]	MO, FR, fibrosis, MA	Binary scale: MO, FR, fibrosis, MA	VL, VI, VM, RF, FL, AL, AM, Pe, Gr, Gl, ST, SM, BF, Sa	-
Van De Vlekkert et al. [25]	MO, SCO, FO FR	SQS: MO, SCO, FO (0–3-point scale). Binary scale: FR	Upper legs or arms	-
Zheng et al. [26]	MO, FR	SQS and modified Mercuri scale: FR and MO (0–5 scale)	FR, VL, VI, VM, Sa, Gr, AL, AM, BF, ST, SM, GM	-
Tasca et al. [27]	MO, FR	MO and FR: typical, consistent, or non-typical pattern	Upper and lower limbs	-
Pipitone et al. [28]	MO	SQS: MO (0–17 scale)	GM, QF, VL, iliopsoas, VM, TFL, RF, Sa, Gr, Pe, AM, AL, BF, SM, ST	ICC=0.78
Barsotti et al. [29]	MO	SQS: MO (0–3-point scale), and binary scale	FG, VM, VL, VI, Sa, Gr, AL, AM, BF, ST	IoB=0.95, InB=0.93
Andersson et al. [30]	MO, FR, FO	SQS: MO (0–3-point scale) and FR (0–5-point scale). Binary scale: FO	Anterior, posterior, and medial compartments and involved in hip flexion/abduction and knee/flexion/extension	-
Pinal-Fernandez et al. [31]	MO, FR, MA, FO	SQS: MO, FR, FO, IFO (0–3-point scale)	GM, OE, OI, RF, VM, VI, VL, Sa, Gr, AL, AM, SM, ST, BF	-
Villa et al. [32]	MO, soft-tissue, perifascicular edema, FR	SQS: MO and FR (0–4-point scale)	Pelvic girdle, Th, leg, shoulder girdle	-
Zhao et al. [33]	MO, FR	SQS: MO (0–5-point scale) and FR (0–5 modified Mercuri scale)	VI, VM, VL, RF, BF, ST, SM, AL, AM, Sa, Gr	-
De Lorenzo et al. [34]	MO, FR, MA, FO	Binary scale: MO, FR, MA and FO	GM, OE, OI, RF, VM, VI, VL, Sa, Gr, AL, AM, SM, ST, BF	-
Ran et al. [35]	MO	Quantitative: T2 mapping with mean relaxation time values from ROIs	VM, VL, VI, RF, AM, AL, ST, SM, BF	-
Dahlbom et al. [36]	MO, FR, MA	SQS: MO, FR, MA (0–4-grade scale)	TA, VL (right leg), biceps brachii (left arm)	-
de Souza et al. [37]	MO, FR, MA	SQS: MO, FR, MA (0–4-grade scale)	Th	-
Ukichi et al. [38]	SC adipose tissue, fascia, muscle	Binary: structures with HIS (SC adipose tissue, fascia, muscle), distributions of HIS areas in muscle (diffuse, patchy, peripheral), patterns of HIS (honeycomb, foggy, strong HIS)	Th	IoB *k* = 0.66–0.98
Aoki et al. [39]	MO, FO	SQS: MO (0–3-point scale) Binary: MRI abnormalities	Bilateral Th or unilateral upper arm	-
Marty et al. [40]	Muscle area, FR	Quantitative: T1, T2 and fat fraction maps.	VL, VM, VI, RF, SM, ST, BF	-
Wang et al. [41]	MO, MA	Quantitative: T2 maps	VM, VL, RF, VI, AL, AM, BF, SM, ST	-
Day et al. [42]	MO, FR, MA	SQS: MO, FR, MA (0–3-grade scale)	P, AL, AM, anterior and posterior Th; ADLL, PDLL	IR reliability: low for several compartments, particularly when assessing edema (*k* = 0.30–0.58); more favorable (*k* > 0.6117) for anterior Th atrophy and FR in the AM, AL, posterior Th, ADLLs and PDLLs
Lassche et al. [43]	MO, FR	Quantitative: FR by manually tracing Binary scale: MO	VL, TA	-
Müller et al. [44]	MV	Quantitative: semi-automated and manually annotated	Thigh and lower leg	Total MV: semi-automated: 2613cm^3^; manual:2594cm^3^
Zhao et al. [45]	MO, FR	SQS: FR (0–5 scale, the modified Mercuri scale); MO (0–5 scale).	GM, VI, VM, VL, RF, BF, ST, SM, AM, AL, Sa, Gr	-
Ansari et al. [46]	FR, MO, MV	Quantitative: MRI indices: the parameters were assessed from the mean pixel intensity of the normalized histogram	VL, VM, VI, RF, SM, ST, BF, Sa, Gr, AL, AM, TA, extensor and flexor HL, extensor and flexor DL, fibularis brevis and longus, TP, medial and lateral Ga, soleus	-
Araujo et al. [58]	MO, FF	Quantitative: T2 relaxation parameters from ^1^H-MRS and T2-mapping. Fat fraction estimated from MRS	TA, Ga, S, VL, VM, RF, Sa, Gr, AL, AM	-
Farrow et al. [47]	MO, MV, FR, SC fat, fascial tissue	SQS: MO, MV, FR (0–4-point scale)	SM, ST, BF, RF, VM, VL, VI	-
Lee et al. [48]	MO, FO, FR	SQS: FR (the Mercuri classification) Binary scale: MO, fascial edema	Th (gluteal, anterior, medial, posterior compartments)	-
Reyngoudt et al. [57]	FF	Quantitative: ΔFat % derived from three segmentation strategies using Dixon and multi-spine echo sequences; ROIs defined for individual muscles, muscle groups, and global segments via custom code	Thigh and lower leg	-
Zhang et al. [49]	MO, FO, SCO, FR	SQS: MO: 0–4-grade scale, and FR: 0–5-grade scale (Mercuri)	TFL, GM, OE, OI, QF, Sa, RF, VI, VM, VL, Pe, AL, AM, BF, SM, ST	-
Oto et al. [50]	MO, FR	Binary scale: MO, FR. Classification of edema in fascial or intramuscular pattern	Deltoids, biceps, triceps, FR, VM, VL, VI, AM, AL, Gr, Sa, BF, SM, ST, GM	IoB=0.644
Laurent et al. [52]	MV, FR, MO, SC, inter and intramuscular adipose tissue	Quantitative: Parameters determined from the 2D proton density-weighted images	FR, VL, VI, VM, Sa, ST, SM, BF, Gr, AM, AL	-
Fionda et al. [51]	MO, FR	SQS FR (0–40 scale (Fisher classification). Binary scale: MO	39 muscles (lower body, including pelvis girdle and lower limb muscles), 18 muscles of upper body, including scapular girdle and arms	IR agreement: *k* = 0.87
Barsotti et al. [53]	MO, MA, FR, FO, SCO	SQS: MO and MA (Likert 0–4-grade scale), and FR (0–3-grade scale) Qualitative analysis.	-	IoB=0.91, InB=0.86
Kimura et al. [54]	MO, FR, FO, MA	Binary scale: MO, FR, FO and MA	OI, OE, GM, AL, AM, QF, RF, VM, VI, VL, Sa, Gr, SM, ST, BF	-
Gorijavolu et al. [55]	MO (intensity, extension), extent of fascial edema, MF, FR	SQS: MO score for each muscle was calculated by multiplying MO intensity with the MO extent. For each of the t-MRI variables, a cumulative score was obtained by adding the scores of all 15 muscles in each Th. Cumulative score from both the Th for every MRI variable were averaged and taken as the final score	GM, OE, OI, AL, AM, adductor brevis, Sa, RF, VL, VM, VI	ICC>0.85; MO 0.97, fascial edema 0.92, MA 0.87, FR 0.97
Reyngoudt et al. [56]	FF, contractile CSA (cCSA)	Quantitative: FF maps and CSA were reconstructed from GRE images using the three-point Dixon method	EDL, TA, TP, PL, S, Ga, VL, VI, VM, RF, Gr, Sa, AL, AM, BF, SM, ST	-

ADLL: anterior distal lower limb; AL: adductor longus; AM: adductor magnus; BF: biceps femoris long head; CSA: cross-sectional area; CI: confidence interval; DL: digitorum longus; HIS: high signal intensity; IFO: interfascial oedema; FF: fat fraction; FO: fascial oedema; FR: fatty replacement; Ga: gastrocnemius; Gl: gluteus; GM: gluteus maximus; GRE: gradiente-recalled echo; Gr: gracilis; HL: hallucis longus; ICC: intraclass correlation coefficient; InB: intraobserver agreement; IoB; interobserver agreement; IR: inter-rater; QF: quadratus femoris; MA: muscle atrophy; MO: muscle oedema; MRS: magnetic resonance spectroscopy; MV: muscle volume; OE: obturator externus; OI: obturator internus; PDLL: posterior distal lower limb; PL: peroneus longus; Pe: pectineus; RF: rectus femoris; Sa: sartorius; SC: subcutaneous; SQS: semi-quantitative scoring; SM: semimembranosus; ST: semitendinosus; STIR: short tau inversion recovery; S: soleus; TA: tibialis anterior; Th: thighs; TP: tibialis posterior; TSL: tensor fasciae latae; VAS: visual analogue score; VI: vastus intermedius; VL: vastus lateralis; VM: vastus medialis.
