# Supplementary material for: Magnetic resonance imaging for adult idiopathic inflammatory myopathies: A scoping review of protocols, grading systems and applications
Source: Semin Arthritis Rheum. Author manuscript; Available in PMC 2026 Apr 4. (PMC13050306; doi:10.1016/j.semarthrit.2025.152865)
Supplement: Supplementary tables [file NIHMS2147666-supplement-Supplementary_tables.docx]

Table 1 (Supplement). Whole body muscle magnetic resonance imaging (objectives and conclusions)

| *Reference* | *Objectives* | *MRI-related Results/Conclusions* |
| --- | --- | --- |
| Cantwell et al. (7) | To describe the use of WB-MRI STIR MRI in the diagnosis and assessment of IIM | Radiologists correctly differentiated DM, PM and IBM. Key discriminators: subcutaneous edema (DM), patchy muscle edema (DM), diffuse muscle edema (PM), forearm, thigh and calf involvement (IBM). As such, WB-MRI may discriminate between IIM subtypes. |
| Filli et al. (8) | To evaluate whether omission of trunk imaging affects diagnostic accuracy of WB-MRI in IIM | Compared diagnostic accuracy of a standard WB-MRI protocol with a restricted WB-MRI protocol (omission of trunk imaging) in patients with IIM. Omission of trunk does not affect diagnostic accuracy of WB-MRI in IIM. |
| Elessawy et al. (9) | To evaluate the use of WB-MRI in characterization of IIM | Site and distribution of radiographic changes in IIM reported. A strong correlation between muscle edema and muscle disease activity observed. WB-MRI captures the burden and distribution of muscle edema, fascial edema, subcutaneous edema in IIM. |
| Huang et al. (4) | To evaluate the value of WB-MRI in diagnosing muscular and extramuscular lesions in IIM | 81% IIM patients had muscle edema, most commonly thigh (81%), pelvis (73%), shoulders (72%), lumbar muscles (66%), neck (60%). WB-MRI had a higher positive rate than CK or EMG.  WB-MRI was 74.3% sensitive and 88% specific for ILD. Malignancy was detected in 4.8%. |
| Guimaraes et al. (10) | To assess MRI findings in patients with IBM and correlate them with clinical and functional parameters | 100% IBM patients had muscle fatty replacement, most commonly vastus lateralis (83%), flexor digitorum profundus (FDP, 80%), medial gastrocnemius (MG, 100%). 83% had muscle edema, most frequently MG and FDP. Thus, IBM presents with a distinctive pattern of muscle involvement on WB-MRI.  There was a statistically significant correlation between disease duration and number of muscles infiltrated by fat (r = 0.65; p = 0.04). Fatty replacement correlated with reduced muscle strength in affected muscles (*r* = –0.60; *p* = 0.04) and worse functional outcomes as measured by the Modified Rankin Scale (*r* = 0.48; *p* = 0.03). Similarly, muscle atrophy was inversely associated with muscle strength (*r* = –0.58; *p* = 0.04). No significant associations were found between edema patterns or serum CK levels and clinical or functional measures (*p* > 0.05). |
| Milisenda et al. (11) | To compare MRI findings with clinical features | Muscle edema correlates with CK and histology in DM. |
| Faruch et al. (12) | To compare the ability of DWI-WB-MRI and STIR-WB-MRI to detect muscle edema in IIM | DWI sequences detected more muscle than STIR and hence appear more sensitive. There was a moderate correlation between CK and muscle edema. |
| Karino et al. (13) | To explore risk factors for RP-ILD in DM using WB-MRI | Muscle edema positively correlated with CK and negatively correlated with strength (MMT average). Proximal lower limb muscles more affected than trunk or upper limb. Gluteal muscle edema was common, including in CADM.  A high fascial edema/muscle edema ratio was an independent risk factor for RP-ILD. |
| Landon-Cardinal (19) | To perform WB-MRI in order to analyze (i) the pattern and the severity of muscle damage in IMNM and (ii) its relationship with serological status and the clinical course of the disease | IMNM is associated with severe axial and pelvifemoral muscle damage. Disease duration is an important predictor of muscle damage. IMNM and IBM patients have a comparable damage burden. |
| Fabry et al. (14) | To assess the capability of a deep learning tool to discriminate between FSHD1 and IIM using WB-MRI | Radiologists correctly differentiated FSHD1 from IIM in 87.5-95% cases. Thus WB-MRI may discriminate myopathies. |
| Matsuda et al. (15) | To describe inflammatory changes in skeletal muscles of IIM patients using WB-MRI | Extensive muscle edema was observed in ASyS and IMNM. Pelvic, abdominal muscles preferentially involved in anti-mitochondrial antibody positive myositis. The thigh preferentially involved in IBM. Muscle edema correlated with CK but not strength (measured as MRC sum). |
| Walter et al. (16) | To prospectively compare US and WB-MRI for detection of muscle abnormalities in IIM | WB-MRI is more sensitive than US for detection of muscle abnormalities in the early phase of IIM. WB-MRI reveals subclinical muscle abnormalities. The number of edematous muscles of WB-MRI decreased non-significantly over time. The MRI oedema sum score negatively correlated with MMT13. |
| Cavalcante et al. (17) | To analyze WB-MRI features in IM-Mito compared with IBM | WB-MRI imaging features were similar between IM-Mito and IBM, with the highest mean degree of fatty replacement within the quadriceps and medial gastrocnemius. There remains uncertainty whether these two conditions are part of the same clinical spectrum. |
| Zierer et al. (18) | To compare WB-MRI findings across a range of IIM subtypes | Severe muscle fatty replacement was characteristic of IBM, with consistent involvement of quadriceps femoris, sartorius, medial gastrocnemius and flexor hallucis longus. PM-Mito, PM, ASyS, overlap myositis, and IMNM displayed only modest fatty replacement with no consistent pattern. Muscle edema was mainly confined to lower extremities in IBM and PM-Mito, whereas PM, ASyS, overlap myositis, and IMNM displayed broader muscle involvement. Quadriceps muscle means fat fraction correlated with disease duration for IBM. There was no correlation between mean fat fraction and CK. |

CK: serum creatine kinase; EMG: electromyography; DM: dermatomyositis; DWI: diffusion-weighted; FSHD: fascioscapulohumeral muscular dystrophy; IBM: inclusion body myositis; IIM: idiopathic inflammatory myopathy; ILD: interstitial lung disease; IVIG: intravenous immunoglobulin; MRI: magnetic resonance imaging; RP: rapidly progressive; WB: whole body; STIR: short tau inversion recovery; US: ultrasound.

Table 2 (Supplement). Dedicated body-part muscle magnetic resonance imaging (objectives and conclusions)

| *Reference* | *Objectives* | *MRI-related findings and conclusions* |
| --- | --- | --- |
| Dion et al. (20) | To develop diagnostic imaging criteria for PM and IBM | Despite some overlap in MRI findings between the two diseases, MRI was useful for distinguishing PM from IBM. CK levels did not correlate with the presence of muscle atrophy or inflammation on MRI. CK levels correlated negatively with the extent of muscle fatty replacement. No correlation was found between muscle strength and the presence of fatty replacement, inflammation, or atrophy on MRI. |
| Tomasová Studynková et al. (21) | To evaluate the relationship of MRI of thigh muscles to clinical and histological parameters in patients with IIM | Muscle biopsy guided by MRI finding contains more inflammatory cells than the biopsy taken from MRI non-affected sites. However, some inflammatory changes were detected in muscles which look unaffected on MRI scan. Muscle edema on MRI scans improved in most patients after 2-6 months of treatment, but the histologically detected inflammation does not change. Clinical activity scores correlated with the intensity and extent of radiographic muscle edema at initial MRI and with extent and total burden of radiographic edema at second MRI. CK correlated with total burden of muscle edema at first MRI but not at follow-up MRI. There was a somewhat better correlation of muscle involvement assessed by MRI in patients with DM than in PM. |
| Degardin et al. (22) | To assess MRI in the diagnostic workups of muscular dystrophies and IIMs by describing the topography of muscle involvement | A specific pattern of muscular involvement was established for each muscular disease. Edema preceded fatty degeneration and was not specific for IIM. |
| Cox et al. (23) | To analyze whether MRI of upper and lower limbs is of additional value in the diagnostic work-up of sIBM | 100% IBM patients had muscle fatty replacement; 78% had muscle edema; 94% had muscle atrophy. Most frequently affected muscles: FDP, vastus, rectus femoris, gastrocnemius. Asymmetric involvement in 44%. Thus, IBM presents with a distinctive pattern of muscle involvement on MRI. Fatty replacement correlated with disease duration, incapacity and weakness. |
| Miranda et al. (24) | To compare MRI in newly diagnosed PM and DM | Muscle edema was found in significantly more muscle compartments in DM versus PM, and mainly in the proximal region of the muscles. Muscle fatty replacement was found predominantly in PM. Partial fat replacement area occurred mainly in the medial and distal regions, whereas total fat replacement area occurred mainly in the distal muscles. DM and PM had different characteristics on MRI, despite sharing many clinical characteristics. |
| Van De Vlekkert et al. (25) | To assess the diagnosis of muscle MRI as a triage test before muscle biopsy and as an add-on test in patients with a presumed diagnosis of subacute-onset IIM | Patients with IIM myositis typically shows a symmetrical distribution of high signal intensity on MRI scans, especially in the quadriceps, adductor, and hamstring muscles. MRI had a sensitivity of 91% and specificity of 69% for diagnosing myositis. Using MRI as a pre-biopsy triage tool or an add-on test significantly increased diagnostic accuracy. Using MRI to select the biopsy site reduced the false-negative rate (FNR) from 23% to 19%. When used as an add-on test for cases with negative biopsy results, MRI reduced the FNR further to 6%. This improvement suggests MRI’s utility in detecting inflammatory changes even in cases where biopsy is inconclusive. Both MRI and muscle biopsy are recommended in patients suspected of having IIM. |
| Zheng et al. (26) | To evaluate muscle MRI changes and the role of MRI in monitoring therapy in patients with myopathy associated with anti-SRP | MRI of the thigh muscles shows a distinct pattern of edema and fatty replacement in patients with anti-SRP myopathy. The degree of muscle edema did not correlate with CK levels or MYOACT scores. The degree of fatty replacement correlated with disease duration. There was no correlation between strength and edema or fatty replacement, however a combined fatty replacement and edema score correlated negatively with strength. Patients with marked fatty replacement were refractory to therapy. |
| Tasca et al. (27) | To define the pattern of muscle MRI in IBM and to assess its accuracy in differentiating IBM from other myopathies that overlap with it clinically or pathologically | A characteristic pattern of muscle involvement in IBM was defined as a ‘melted’ appearance of the distal quadriceps above the knee, along with other supporting criteria. This pattern was able to distinguish IBM from non-IBM with 95% accuracy. Muscle MRI may be particularly helpful in patients with early disease or who lack the classical IBM histopathology. |
| Pipitone et al. (28) | To assess which thigh muscle groups are preferentially inflamed in DM and PM | Compared with PM, DM affects more thigh muscles, except those of the posterior compartment, which are equally involved in both disorders. |
| Barsotti et al. (29) | To describe MRI in DM/PM, and correlations between MRI and disease activity | Muscle edema correlated with CK values and PGA. Muscle edema correlated with MMT8 in patients without fatty replacement. Among the 16 patients who underwent follow up MRI at a mean of ﻿49.9 +/- 47.96 months, changes in the degree of MRI edema were not always accompanied by similar changes in CK, PGA, or MMT8 values. Thus, MRI may provide additional information that complements clinical and biochemical examinations. |
| Andersson et al. (30) | To compare MRI between ASSD and control | ASSD patients had more muscle edema, fascial edema, fatty replacement and muscle volume reduction than age and sex-matched control. Muscle MRI changes in ASSD were most common in the posterior compartment followed by the anterior and medial compartments. CK correlated with total muscle edema scores. Strength negatively correlated with total damage scores (a composite of fatty replacement and volume reduction) but there was no association with edema. Total damage was significantly correlated with age and disease duration. Patients with ongoing steroid treatment had lower edema scores; there was no relationship between steroid treatment and muscle atrophy or damage. MRI as an independent tool in the assessment of ASSD-related myositis; particularly in cases with normal CK. |
| Pinal-Fernandez et al. (31) | To define the pattern of muscle involvement in patients with IMNM relative to those with other IIM and to compare patients with IMNM with different autoantibodies | Compared with patients with DM or PM, IMNM is characterized by more widespread muscle involvement. Anti-SRP-positive patients have more severe muscle involvement than anti-HMGCR positive patients. IMNM had more extensive edema than those with either PM or DM. IMNM showed a trend towards more atrophy and fatty replacement than those with either PM or DM. IMNM showed a trend towards having more asymmetry in the percentage of muscles showing fatty replacement than those with DM or PM. Patients with anti-SRP showed more asymmetry than those with anti-HMGCR for all the MRI features. CADM showed the least extensive muscle involvement by MRI. IBM showed greater asymmetry in the percentage of muscles with atrophy compared with PM or DM. IBM did not show a significant association between edema and atrophy or fascial edema, while DM edema was more correlated with fascial edema than in the rest of the clinical group. DM showed a trend towards more asymmetry in fascial edema compared with all the other groups. The positive predictive value of patterns of muscle involvement on thigh MRI for distinguishing IIM subtypes was suboptimal, however the negative predictive value was excellent for IBM (94.7%), IMNM (93.1%) and very good for DM (88.3%). |
| Villa et al. (32) | To describe the clinical and histological features of 5 patients affected with IMNM, as well as their post-treatment outcomes, and to show a new MRI pattern for IMNM recognition that may be helpful in early diagnosis | MRI imaging reportedly shows involvement of the dorsal muscle groups of both the thighs. A new pattern recognition involves also the medial gastrocnemius and, at the arm level, triceps and deltoid followed by infraspinatus and subscapularis. Presence of inflammation of the subcutaneous tissues and of muscularis fasciae of both arms and legs. As such, patients affected with IMNM may exhibit a wider systemic inflammatory response that is not limited to skeletal muscles. Post-treatment (timeframe not reported) MRIs revealed complete resolution of edema and unchanged fatty replacement grades. |
| Zhao et al. (33) | To determine whether MRI could be used for differential diagnosis between MADD and IMNM | There was a significant positive correlation between total edema score and CK level. Total fatty replacement score was positively correlated with onset age and negatively correlated with muscle regeneration. Different patterns of muscle involvement on MRI can help differentiate MADD and IMNM. |
| De Lorenzo et al. (34) | To define the clinical features of patients with anti-PM/Scl (75 or 100) autoantibodies at disease onset and during the course of disease and compare them to patients with other forms of myositis | Thigh MRI revealed muscle edema less frequently in anti-PM/Scl-positive patients (39%) compared to all the other myositis control groups (71% in DM, 76% in AS, and 90% in IMNM). |
| Ran et al. (35) | To explore the T2-mapping signal characteristics of the thigh muscles in patients with DM/PM and to investigate the correlation between thigh muscle T2 values, clinical parameters, and serum CK | The T2 value of all edematous muscles was greater on average than that of the  unaffected muscles of the DM/PM patients and the muscles of healthy volunteers. A statistically significant negative correlation between mean T2 values and muscle strength was observed. However, there was no statistically significant correlation between the mean T2 values and CK. T2 mapping is not only quantitatively used for subclinical muscle involvement in DM/PM, but also used to demonstrate the severity of damaged muscles in DM/PM. |
| Dahlbom et al. (36) | To assess the temporal relation of inflammatory and degeneration changes in IBM through a cross-sectional study | Muscle strength correlated significantly with atrophy observed in MRI. The vastus lateralis, often the weakest muscle, showed the most severe atrophy. Muscle edema was more prevalent in the vastus lateralis, but inflammatory cell infiltration was most pronounced in the biceps brachii. The present study could not provide an answer to the question which comes first, inflammation or degenerative changes. |
| de Souza et al. (37) | To analyze the muscle damage measured by late MRI in IMNM | MRIs were performed late following a treat-to-target treatment protocol (median disease duration 37.9 months) and revealed mild muscle fatty replacement in six patients (46.1%) and moderate fatty replacement in only two patients (15.4%). An early treat-to-target approach could reduce the odds of long-term muscle damage. |
| Ukichi et al. (38) | To define the characteristic findings on MRI of skeletal muscles in DM relative to those in patients with other IIMs and to assess their diagnostic performance in DM | Characteristic MRI findings in DM patients were observed and consisted of subcutaneous edema, fascial edema and muscle edema with a peripheral distribution and/or honeycomb pattern. These patterns discriminated DM from other IIM subtypes with a sensitivity of 72.2% and specificity of 88.5%. Patients with myositis specific or myositis associated antibodies were more likely to exhibit fascial edema and less likely to exhibit muscle edema with a foggy pattern. |
| Aoki et al. (39) | To correlate among muscle pathology, needle EMG, and muscle MRI in different IIMs | Muscle MRI was abnormal in 42 (86%) of patients. Of those who had MRI examinations and muscle biopsy, 75% had MRI abnormalities in the biopsied muscles. EMG changes (fibrillations and positive sharp waves) correlated with bright intramuscular T2 signals. There was no difference in intramuscular T2 changes between patients who had been treated with steroids and those who were steroid-naïve. |
| Marty et al. (40) | To investigate the response of skeletal muscle global T1 under different physiological and pathological conditions using an inversion-recovery radial T1 mapping sequence | Muscle T1 variations are a sensitive indicator of structural changes in skeletal tissues related to physiological events (exercise) as well as pathology in the setting of neuromuscular diseases. For an equivalent fat content, there was a significant increase of T1 in IBM patients compared to those with Becker’s muscular dystrophy. Global T1 changes related to muscle could be complementary to more conventional variables such as FF and T2. |
| Wang et al. (41) | To investigate whether the edema of thigh muscle in DM/PM can be quantitatively assessed by a novel accelerated T2 mapping technique - GRAPPATINI | GRAPPATINI T2 mapping could distinguish between IIM and healthy controls and is more sensitive than conventional MRI. GRAPPATINI shortened the procedure time compared to conventional methods. GRAPPATINI-generated T2 values of DM/PM thigh muscles positively correlated with serum CK, ALT and LDH levels. |
| Day et al. (42) | To assess the burden, distribution, and evolution of muscle inflammation and damage on MRI among subtypes of IIM | Patients with IMNM and IBM have characteristic patterns of muscle MRI abnormalities that may allow them to be differentiated radiologically from other IIM subtypes. There was no correlation between muscle edema and strength or disease activity visual analogue scales. CK correlated with muscle edema in certain muscle groups. There were no correlations between cumulative prednisolone dose and MRI changes. 26 patients underwent serial muscle imaging at a median elapsed time of 478 days. Of these, total fatty replacement and total atrophy scores increased in 50-62%. However, a substantial minority of non-IBM demonstrated improvement in total fatty replacement and total atrophy on serial imaging. As such, muscle damage in non-IBM IIM may be reversible. |
| Lassche et al. (43) | To investigate correlations between muscle MRI abnormalities and histopathological severity in healthy controls and patients with muscle disease | Total quadriceps muscle cross-sectional area was reduced in IBM versus control, while the amount of fatty replacement was increased. As a result, contractile cross-sectional area was reduced by 53% in IBM. Quadriceps strength correlated with the contractile cross-sectional area. In muscle disease, fatty replacement on MRI correlates moderately with muscle histopathology. |
| Muller et al. (44) | To develop a method for semi-automated segmentation of muscle MRI datasets | Muscle volumes were quantified using semi-automated versus manual segmentation. Muscle volumes and ratios of thigh/lower leg volume were lower in myopathy patients than in controls. In myopathy patients, the strength of knee extension showed a strong positive correlation with the thigh muscle volume. |
| Zhao et al. (45) | To evaluate factors associated with refractory ANM-SRP and perform serial MRI scans over an extended period, specifically at 3, 6, 12, 18, and 24 months, to monitor the progression of fatty replacement and edema | Patterns of muscle involvement were similar between patients with and without anti-SRP antibodies. Thigh MRI follow-up showed an increasing rate of fatty replacement and reducing rates of edema. Early thigh muscle fatty replacement was a key predictor of poor treatment response. Specifically, a higher degree of fatty replacement in the first three months was significantly associated with refractory disease. Edema changes did not correlate with treatment response. Early MRI assessments can effectively monitor disease progression and guide treatment adjustments. |
| Ansari et al. (46) | To describe muscle MRI in patients with IBM | Fatty replacement in individual muscles of IBM patients is heterogeneous in terms of proximal-to-distal gradient. The whole muscle fat fraction in the thighs and lower limbs negatively correlated with the Inclusion Body Myositis Functional Rating Scale and lower limb strength. There was no correlation between fatty replacement and CK or disease duration. |
| Araujo et al. (58) | To investigate the multiexponential behavior of the water T2-relaxation in the skeletal muscle of neuromuscular diseases patients, aiming to identify more sensitive and specific biomarkers of disease activity | T2-mono was elevated in patients (P<0.05) but could not distinguish IBM from DMD. While 79% of IBM data presented a biexponential behavior, this was only 16% and 10% for DMD and control data, (P<0.05). All T2 spectra presented an intermediate-T2 peak characterized by an elevated T2 in patients (P<0.05) and by a relative fraction that was abnormally smaller in IBM patients (P<0.05). A long-T2 peak was exclusively observed in IBM patients. T2 spectra provided more sensitive and specific markers of disease presence than the T2-mono, and allowed distinguishing IBM from DMD patients. This must reflect distinct predominant pathological alterations between these diseases, suggesting that these markers provide additional pathophysiological/histopathological information that are missing from T2-mono. |
| Farrow et al. (47) | To assess whether MRI-based measurements of T2, fat fraction, diffusion tensor imaging, and muscle volume can detect differences between the muscles of myositis patients and healthy controls, and to identify how they compare with MRI diagnosis; to investigate the utility of MRI as an indicator of active disease and muscle damage in DM and PM compared with controls | Quantitative MRI measurements can detect differences between myositis patients and controls. Changes in the muscles of myositis patients, undetected by visual, semi-quantitative scoring, can be detected using quantitative T2 measurements. MRI T2 values may be useful for the management of myositis patients. Muscle strength correlated with MRI T2, muscle volume and fat fraction, depending on muscle group examined. |
| Lee et al. (48) | To describe the imaging features of statin-associated anti-HMGCR myopathy on thigh muscle MRI | Statin-associated anti-HMGCR myopathy commonly demonstrates bilateral symmetrical pan-compartmental edema on thigh muscle MRI, with anterolateral predilection in the anterior compartment, and greater involvement of the semimembranosus with relative sparing of the short head of the biceps femoris in the posterior compartment. These observations can contribute to the diagnosis of statin-associated anti-HMGCR myopathy. |
| Reyngoudt et al. (57) | Determine the best strategy for evaluating disease progression | Global muscle segmentation gave high SRMs for ΔFat% in thigh and lower leg for IMNM and only in thigh for IBM.  Global muscle segment Fat% showed to be sensitive to change in most investigated neuromuscular disorders. As compared to individual muscle drawing, it is a faster and an easier approach to assess disease progression. The use of individual muscle ROIs, however, is still of interest for exploring selective muscle involvement |
| Zhang et al. (49) | To evaluate MRI changes to define muscle-lesion specific patterns in patients with ASSD, and compare them with those in other common IIM subtypes | Thigh MRI in ASSD exhibited frequent myofascial edema. ASSD edema patterns resembled those of DM more than those of IMNM. Bilateral asymmetry, adductor-muscle relative sparing and remarkable myofascial edema of tensor fasciae latae were the most characteristic in ASSD.  The non-Jo-1 subgroup tended to be more bilaterally symmetric.  When treatment-naive and the treatment-experienced ASSD patients were compared, there was no statistical differences in MRI findings of muscle edema, myofascial edema, subcutaneous-tissue edema or fatty replacement. |
| Oto et al. (50) | ﻿To elucidate the relationship between MRI findings and ILD prognosis and development in anti-MDA5+ DM | Areas of high signal intensity on muscle MRI were present in 53.5% of patients with anti-MDA5 antibody-positive DM, even among some patients without muscle weakness (amyopathic patients). Presence of intramuscular lesions were associated with a favorable prognosis, with higher survival rates and less extensive lung involvement. No correlation between muscle MRI changes and CK was observed. |
| Fionda et al. (51) | ﻿ To investigate muscle MRI in IMNM as outcome measure for disease activity, severity, progression, response to treatment, and to better characterize the pattern of muscle involvement | IMNM patients with early disease had 38% STIR + muscles, treated patients had 15% STIR + muscles. Pelvic girdle (32%) and thigh (34%) muscle edema was more common than leg muscle edema (12%). No difference in total % muscle edema was observed across serological groups. Higher T1 score (fatty replacement) correlated with late treatment start and delayed treatment with IVIG. Fatty replacement was most severe in lumbar, gluteal, adductor and hamstring muscles. 22% of STIR + muscles showed fatty replacement on serial imaging. Higher STIR% at baseline correlated with a higher risk of fatty replacement at follow-up. %muscle edema correlated with CK. Changes in CK over time correlated with changes in STIR positivity. The % of fatty replacement and a lower change in %STIR at follow up correlated with weakness. |
| Laurent et al. (52) | To evaluate changes in muscle structure and composition using a comprehensive multiparameter set of quantitative MRI measures and to assess construct validity and responsiveness of quantitative MRI measures in IBM | Abnormal muscle quality measured by qMRI is associated with reduced physical performance, including strength, mobility and function. Inflammation may play a role in triggering fat replacement into muscle. Significant changes were observed after 1 year for the majority of qMRI measures, but not for physical performance tests. Only the decrease in total muscle volume was weakly (albeit significantly) correlated with a decrease in muscle strength, highlighting the greater sensitivity of muscle qMRI over more conventional measures of physical performance. MRI provides valid and responsive measures that might prove valuable in sIBM experimental trials and assessment of muscle pathologic processes. |
| Barsotti et al. (53) | To analyze the potential role of MRI in the differential diagnosis between IIM and muscular dystrophies | A different distribution of muscular involvement between IIM and muscular dystrophy (MD) was identified. Edema was significantly more prevalent in IIM compared with MD in pelvis, anterior and medial thigh muscles. Fatty replacement and muscular atrophy were more prevalent in MD. The probability of IIM increased with higher edema score and decreased with higher atrophy and intramuscular Fatty replacement /substitution scores. |
| Kimura et al. (54) | ﻿ To compare the findings of muscle magnetic resonance imaging (MRI) between anti-signal recognition particle antibody-positive myopathy (anti-SRP myopathy) and anti-aminoacyl-tRNA synthetase antibody-positive myositis (anti-ARS myositis) | Edema within the thigh muscles was observed in most cases across both the anti-SRP and anti-ARS groups, however fascial edema was exclusively identified in the anti-ARS group, particularly among Jo-1 positive patients. Additionally, gluteus maximus muscle abnormalities were significantly more frequent in the anti-SRP group compared to the anti-ARS group. |
| Gorijavolu et al. (55) | To evaluate the relationship of thigh MRI with MMT-8, muscle enzymes and autoantibodies. To determine the causal and mediating factors resulting in poor recovery of MMT-8 in IIM | Baseline MMT-8 negatively correlated with muscle edema, fascial edema and muscle atrophy, but not with fatty replacement. Creatinine kinase and aspartate transaminase positively correlated with muscle edema. Follow-up MMT-8 at median 31 (10-57) months correlated negatively with baseline atrophy and fatty replacement. None of the t-MRI parameters correlated with disease duration. Older age, female sex, longer duration of illness, and absence of anti-synthetase antibodies resulted in lower recovery of skeletal muscle power and that these factors possibly act via pathways of muscle atrophy and fatty replacement. |
| Reyngoudt et al. (56) | To examines the complete quantitative MRI and phosphorus MRS (31P MRS) data from the clinical phase-2b trial of sirolimus and their relationship with muscle function and strength | The *quadriceps* and *gastrocnemius medialis* muscles had the highest FF values, displaying notable heterogeneity and asymmetry, particularly in the *quadriceps*. In the placebo group, the median 1-year FF increase in the quadriceps was 3.2% (*P <* 0.001), whereas in the sirolimus group, it was 0.7% (*P* = 0.033). Both groups experienced a significant decrease in cCSA in the *quadriceps* after 1 year (*P <* 0.001), with median changes of 12.6% for the placebo group and 5.5% for the sirolimus group. Differences in FF and cCSA changes between the two groups were significant (*P <* 0.001). Additionally, significant correlations were observed between FF, cCSA, water T2, and functional and strength outcome measures. |

ASSD: antisynthetase syndrome; ADM: amyopathic dermatomyositis; ANM: autoimmune necrotizing myopathy; CADM: clinically amyopathic dermatomyositis; CK: serum creatine kinase; DM: dermatomyositis; EMG: electromyography; GRAPPATINI: combination between model-based accelerated relaxometry by interactive nonlinear investigation (MARTINI) with generalized autocalibration partial parallel acquisition (GRAPPA); IBM: inclusion body myositis; IIM: idiopathic inflammatory myopathy; IMNM: immune-mediated necrotizing myopathies; LGMD: limb-girdle muscular dystrophy; MADD: multiple acyl-CoA dehydrogenase deficiency; MMT: Manual Muscle Testing; MR: magnetic resonance; MRI: magnetic resonance imaging; MYOACT: Myositis Disease Activity Assessment Visual Analog Scales; PGA: Patient Global Assessment; PM: polymyositis; SRP; signal recognition particle.
